# Cell type matters: competence for alkaloid metabolism differs in two seed-derived cell strains of *Catharanthus roseus*

**DOI:** 10.1007/s00709-022-01781-y

**Published:** 2022-06-13

**Authors:** Manish L. Raorane, Christina Manz, Sarah Hildebrandt, Marion Mielke, Marc Thieme, Judith Keller, Mirko Bunzel, Peter Nick

**Affiliations:** 1 Botanical Institute, Karlsruhe Institute of Technology, Fritz-Haber-Weg 4, 76131 Karlsruhe, Germany; 2 Present Address: Institute of Pharmacy, Martin-Luther-University, Hoher Weg 8, 06120 Halle-WittenbergHalle (Saale), Germany; 3 Institute of Applied Biosciences, Department of Food Chemistry and Phytochemistry, Karlsruhe Institute of Technology (KIT), 76131 Karlsruhe, Germany

**Keywords:** *Catharanthus roseus* seed embryos, Suspension cell strains, Vinca alkaloids, Metabolic competence, Elicitors, Precursor feeding

## Abstract

Since the discovery of the anticancer drugs vinblastine and vincristine, *Catharanthus roseus* has been intensively studied for biosynthesis of several terpene indole alkaloids (TIAs). Due to their low abundance in plant tissues at a simultaneously high demand, modes of production alternative to conventional extraction are mandatory. Plant cell fermentation might become one of these alternatives, yet decades of research have shown limited success to certain product classes, leading to the question: how to preserve the intrinsic ability to produce TIAs (metabolic competence) in cell culture? We used the strategy to use the developmental potency of mature embryos to generate such strains. Two cell strains (C1and C4) from seed embryos of *Catharanthus roseus* were found to differ not only morphologically, but also in their metabolic competence. This differential competence became manifest not only under phytohormone elicitation, but also upon feeding with alkaloid pathway precursors. The more active strain C4 formed larger cell aggregates and was endowed with longer mitochondria. These cellular features were accompanied by higher alkaloid accumulation in response to methyl jasmonate (MeJA) elicitation. The levels of catharanthine could be increased significantly, while the concurrent vindoline branch of the pathway was blocked, such that no bisindole alkaloids were detectable. By feeding vindoline to MeJA-elicited C4 cells, vincristine became detectable; however, only to marginal amounts. In conclusion, these results show that cultured cells are not “de-differentiated”, but can differ in metabolic competence. In addition to elicitation and precursor feeding, the cellular properties of the “biomatter” are highly relevant for the success of plant cell fermentation.

**Supplementary Information:**

The online version contains supplementary material available at 10.1007/s00709-022-01781-y.

## Introduction

Plant metabolites belong to two main groups, namely, the primary metabolites needed for vital cell functions; and the secondary metabolites, which are not needed for the individual cell, but are essential for the organism. These secondary metabolites include not only small molecules that are involved in environmental interactions, but also hormones which modulate metabolism and integrated functions within a multicellular organism (Erb and Kliebenstein 2020). Currently, more than 100,000 of these compounds have been identified from plants (Zhong and Yue 2005). Apart from being important to the plant itself, they are also very beneficial to humans as medicinal compounds against various diseases. More than 50,000 plant species have been used for medicinal purposes (Gómez-Galera et al. 2007). Natural products or their analogues have been instrumental in over 60% of anticancer drugs (Newmann et al. 2003). Some of the most significant examples are vinblastine and vincristine from *Catharanthus roseus* and paclitaxel from the bark of *Taxus brevifolia* and other *Taxus* species.

**Fig. 1:**
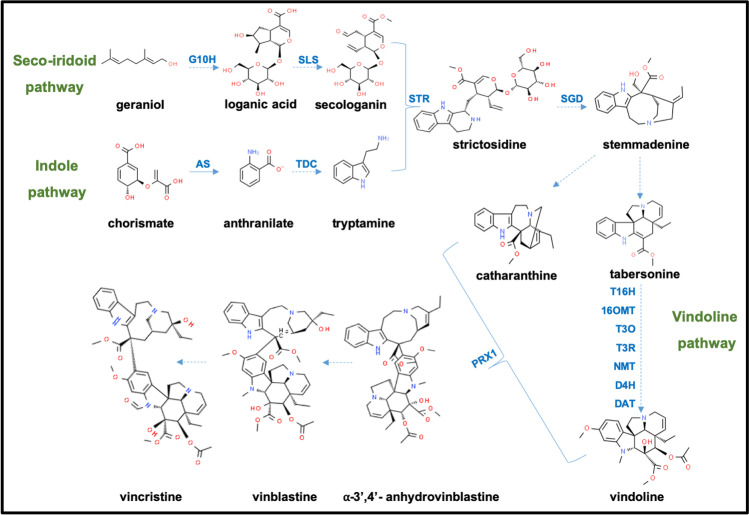
Schematic representation of terpene indole alkaloid (TIA) biosynthetic pathway of *Catharanthus roseus*. The early TIA pathway extends across the seco-iridoid pathway (terpene moiety) and the shikimate pathway (indole moiety). The late TIA pathway refers to all downstream synthesis steps from the condensation of secologanin and tryptamine to form central TIA precursor strictosidine. This is followed by the catharanthine and vindoline biosynthesis pathway. Finally, coupling of vindoline and catharanthine leads to production of vinblastine and vincristine through the vinblastine-vincristine pathway. Solid arrows indicate direct enzymatic reaction and broken arrows represent multiple or uncharacterized reactions. G10H, geraniol 10-hydroxylase; SLS, secologanin synthase; AS, anthranilate synthase; TDC, tryptophan decarboxylase; STR, strictosidine synthase; SGD, strictosidine-β-glucosidase; T16H, tabersonine16 hydroxylase (both the isoforms T16H1 and T16H2); 16-OMT, 16-hydroxytabersonine 16-O-methyltransferase; T3O, tabersonine-3-oxygenase; T3R, tabersonine 3-reductase; NMT, N-methyltransferase; D4H, deacetoxyvindoline-4-hydroxylase; DAT, deacetylvindoline-4-O-acetyltransferase; PRX1, peroxidase 1/anhydrovinblastine synthase

The biosynthetic pathway of TIAs in *C. roseus* is very complex (Fig. 1). More than 50 biosynthetic events are involved, including four different cell types, five different subcellular compartments, and intercellular transport of pathway intermediates, culminating in the synthesis of the final products vinblastine and vincristine (Qu et al. 2019; Ziegler and Facchini 2008). The central intermediate, strictosidine, is formed by the condensation of the indole precursor tryptamine, and the terpenoid precursor secologanin (Courdavault et al. 2014; Thamm et al. 2016). Strictosidine is then converted to stemmadenine, through which various TIAs, such as ajmaciline, catharanthine, or tabersonine, are produced. Tabersonine is transformed through seven well-characterised enzymatic steps into vindoline (St-Pierre et al. 1998; Liscombe et al. 2010; Besseau et al. 2013; Qu et al. 2015). Recent research has improved our understanding of the downstream pathway—with at least 26 identified genes in catharanthine and vindoline production shown to be involved (Qu et al. 2019). Vindoline and catharanthine undergo peroxidase-mediated coupling to form the highly valuable bisindole alkaloids—vinblastine and vincristine (Sottomayor and Ros Barceló 2003).

These anticancer alkaloids generally accumulate in the plant tissue to only very low concentrations. In combination with the increasing demand, the costs of vinblastine and vincristine have sky-rocketed to $1,000,000/kg (Miettinen et al. 2014; O'Keefe et al., 1997). The growing prices have stimulated the search for alternatives to extraction from plant material. Genetic transformation of the plants, as well as the synthetic and/or semi-synthetic routes of production of these metabolites, has been employed with variable and modest success. Only recently, overexpression of *CrTDC* and *CrSTR* in *C. roseus* plants has allowed enhancing vinblastine production fivefold (Sharma et al. 2018). Irrespective of the political controversy about the agricultural use of transgenic plants, extending this transgenic production platform sufficiently to meet the growing demands could be challenging. A synthetic route for molecules of this complexity is also far from practical (Chemler and Koffas 2008). Consequently, strategies for semi-synthesis are currently the favoured route to produce these compounds, often in combination with heterologous systems of production. For instance, Qu et al. (2015) engineered the complete seven-gene vindoline pathway into yeast to produce vindoline from tabersonine, while Brown et al. (2015) achieved de novo production of strictosidine in yeast by introducing 21 genes and 3 gene deletions into the yeast genome. Recent work by Caputi et al. (2018) led to the identification of the last two missing enzymes necessary for conversion of stemmadenine acetate to catharanthine and vindoline. Thus, despite the fact that production of the final products vinblastine and vincristine still has remained elusive, these breakthroughs provide some prospects upon a semisynthetic production of these anti-cancer compounds in heterologous systems.

Plant cell fermentation represents a promising alternative strategy for bio-production of desired compounds. Suspension cells show rapid growth and are often able to recapitulate biosynthetic potencies of plant tissues (Imseng et al. 2014). The cells have the potential to produce large amounts, and like in plant tissues, toxic side effects can be avoided either by sequestering such compounds in the central vacuole or by secreting them to the medium. It is also possible to engineer metabolic pathways by transgenic approaches (Rao and Ravishankar 2002). Despite these advantages, the examples for an economically successful implementation of plant cell fermentation have remained limited. Decades of research have failed providing any significant breakthrough in industrial-scale production of vincristine and vinblastine.

TIA pathway genes can be activated in the context of biotic and abiotic stress (Courdavault et al. 2014). Since many plant stress responses involve the activation of the phytohormonal jasmonate pathway, jasmonates have been successfully employed to mimic wounding, pathogen attack, or herbivores (Wasternack and Hause 2013). In fact, jasmonates have been shown to effectively induce plant secondary metabolites (Naik and Al-Khayri 2016), such that, in the biotechnological context, they are often termed as elicitors (in analogy to microbial inducers of defence, the elicitors in sensu stricto). In *Catharanthus*, jasmonates can induce the expression of TIA biosynthesis and their regulators (Patra et al. 2017; Geerlings et al. 2000; van der Fits et al. 2000), such as the transcription factors *ORCA2* and *ORCA3* (Zhang et al. 2011). On this base, JA elicitation has been employed in *C. roseus* cell strains, hairy roots, and plantlets, and this allowed to induce some steps of the TIA pathway network and to see subsequent increases of ajmalicine, catharanthine, serpentine, and tabersonine (Peebles et al. 2009; Ruiz-May et al. 2009; Shukla et al. 2010; El-Sayed and Verpoorte 2004). However, accumulation of vindoline production in cell and hairy root strains of *C. roseus* has not been achieved, so far (Shukla et al. 2010), leading to the question: which aspect of alkaloid metabolism in real tissue is missing in cell culture?

In the leaf of *Catharanthus*, TIA metabolism is partitioned to different cell types (St Pierre et al. 1999; Ziegler and Facchini 2008), which, upon elicitation, express different branches of the metabolic network. While suspension cells are often interpreted as “de-differentiated”, they usually maintain certain features of their source tissues and, therefore, often differ in their response to exogenous factors such as phytohormones or elicitors (Opatrný et al. 2014). For such cellular differences in the response to external factors, the term “competence” has been coined (Mohr 1972). Even neighbouring cells in a tissue can differ in their metabolic competence, as shown by microirradiation experiments (Nick et al. 1993). Also, in *C. roseus*, different organs have been found to show very different metabolic and transcriptional profiles indicative of differential metabolic competence (Shukla et al. 2006; Laflamme et al. 2001).

To overcome the limitations of plant cell fermentation, it might be rewarding to search for cell strains with different and complementary metabolic competence. While plant cell strains can be potentially generated from any tissue of the plant such as leaf, stem, roots, and seeds, mature embryos are interesting because they should still reflect the full diversity of metabolic competence. In appropriate conditions, explants proliferate into a callus, which can be transferred to a liquid medium for creating suspension strains (Hall 2000; Wilson and Roberts 2012). These “de-differentiated” cells might still retain the metabolic competence of their source tissue. In fact, we show in the current study that two cell strains derived from the mature embryo of *Catharanthus roseus* are not only different with respect to their morphology but also differ with respect to their competence for TIA biosynthesis. By tailoring culture conditions and jasmonate elicitation, we can, in one of these strains, stimulate the accumulation of the TIA precursor catharanthine. By feeding the product of the concurrent pathway, vindoline, we can detect the final product vincristine, albeit at very low level and not in a stable manner. Nevertheless, this study demonstrates that metabolic competence persists in cell culture and is relevant for the accumulation of the desired metabolites.

## Material and methods

### Chemicals

Monoindole alkaloid standards, such as catharanthine, tabersonine, and vindoline, as well as other chemicals, such as ammonium acetate, triethylamine, driselase, and sucrose, were purchased from Sigma-Aldrich (Missouri, USA). Bisindole alkaloid standards like vinblastine and vincristine were purchased from Cayman Chemicals (Michigan, USA). Acetonitrile and methanol were all LCMS grade and purchased from Merck (Darmstadt, Germany). Water was treated in a Milli-Q (Millipore, USA) water purification system. The other chemicals used for growth and elicitation studies, such as Gamborg B5 medium, 2,4-dichlorophenoxyacetic acid (2,4-D), jasmonate (JA), and methyl jasmonate (MeJA), were purchased from Duchefa Biochemie (Haarlem, Netherlands). The dye to stain for cell viability, fluorescein diacetate (FDA), was purchased from Merck Chemicals, Germany; the fluorochrome for mitochondria, MitoTracker Red FM, was purchased from Thermo Fisher Scientific Inc., Waltham, MA, USA.

### Cell strains

The suspension cell strains of *Catharanthus roseus* (L.) G. Don used in the current study, C1 and C4, were provided by Phyton Biotech GmbH (Ahrensburg, Germany) and originated in 2004 from seed embryos of *Catharanthus roseus* plants cultivated in Chile and are maintained as reference in the cryobank of Phyton Biotech GmbH (Ahrensburg, Germany). The cell strains C1 and C4 were derived from cultivars *Catharanthus roseus* Heatwave™ Pink and *Catharanthus roseus* Stardust orchid respectively. These cell strains were well established as suspension cultures, and cultivated in fresh and autoclaved growth media containing Gamborg B5 salts (3.21 g/l), sucrose (30 g/l), and 2,4-D (5 μM), adjusted to pH 5.6 ± 0.03. These strains were sub-cultured weekly, by inoculating 3 g (fresh weight) of filtered cells into the growth medium (50 ml) in 250-ml polycarbonate Erlenmeyer flasks with filter caps (Corning GmbH, Kaiserslautern, Germany). The cells were incubated at 26 °C in the dark on a gyratory platform shaker (Heidolph Instruments GmbH, Germany) at 120 rpm. For experimentation, smaller aliquots were cultivated using 125-ml polycarbonate Erlenmeyer flasks with filter caps, containing 25 ml of medium inoculated with 1.5 g fresh weight of cells.

However, in case of long-term studies, to follow long-term responses, in some experiments, the authors performed a slight modification to the culture setup to understand the phenotypic characteristics of these strains better. The cell strains (C1 and C4) were cultured by inoculating 3 g (fresh weight) of filtered cells into the growth medium (50 ml) in 250-ml Erlenmeyer flasks, and allowed to grow for 15 days continuously without any passaging after 7 days. This experimental approach was repeated in 5 biological replicates.

### Quantitative phenotyping

To characterise the phenotype of these *Catharanthus* cell strains, different parameters of cell morphology and growth were quantified. These included cell size, cell volume, cell density, cell cycle duration, cell fresh and dry weight, packed cell volume (PCV), and cell viability. Individual cells were observed and captured under differential interference contrast, using an Axio Imager Z1 microscope (Zeiss, Jena, Germany), and the images were analysed using the AxioVision software (Rel. 4.8.2; Zeiss, Germany).

Prior to microscopic analysis, a special pre-treatment was required specifically for the C4 cell line which formed very large aggregates in the culture medium. These cells were specifically treated with 0.25% driselase for 20 min at 25 °C. In general, these parameters were followed over the entire cultivation cycle, with each data point representing the average of 500 individual cells from three independent experimental setups.

To measure cell size, microscopic images were captured using the MosaiX-module sampling system (Zeiss, Germany) to avoid sampling bias. The covered area was approximately 2.5 mm^2^ composed of 25 individual images. Cell length and breadth were measured from the central section of the cell using the AxioVision software (Maisch and Nick 2007).

The volume of the cells was estimated on the basis of the cell size measurements approximating shape by a cylinder model (Sakano et al. 1995), i.e. as

$$V=\uppi \times \left(\frac{d}{2}\right)2\times L$$

with *d* is the cell width and *L* is the cell length. Cell density was estimated using a haemocytometer (Fuchs-Rosenthal) under bright-field illumination. To infer doubling time, an exponential model for proliferation was assumed.

$${N}_{t}={N}_{0}\times {e}^{kt}$$

Here, *N*_*t*_ is the cell density at time point *t*, *N*_0_ is the cell density at inoculation, *e* is the Euler constant, and *k* is the time constant. The reference was set after the starting number (*N*_0_) was counted immediately after sub-cultivation of cells in a fresh medium flask.

Cell viability was quantified by staining with fluorescein diacetate (Widholm 1972). Briefly, 0.1% of 5 mg/ml FDA dye was added and viewed directly. Living cells are fluorescent green, since cytoplasmic esterases cleave the non-fluorescent FDA into the fluorescent product fluorescein. Dead cells lacking this enzyme activity emit no fluorescence. The FDA signal was examined by an AxioImager Z.1 microscope (Zeiss, Jena, Germany), using the filter set 38 HE (excitation: 470 nm, beamsplitter: 495 nm, and emission: 525 nm, Zeiss).

Both cell strains were also studied for their growth potential. As parameters fresh weight, dry weight, and packed cell volume (PCV) were measured at the end of a cycle at day 7 after sub-cultivation, the medium was removed from the cell suspension by using Whatman filter no.1 (Whatman, Germany) under vacuum filtration (Vacuum pump ME 4 NT, Vacuubrand GmbH, Germany). The drained cell material obtained was weighed (fresh weight) to determine the growth index of these cell strains at day 7, by dividing it by the value for the initial inoculum. Furthermore, these filtered cell materials were dried in a drying oven at 60 °C for 3 days to obtain dry weight. As an alternative parameter, we estimated PCV as a quick and cost-efficient readout for growth that depends on cell volume as well as cell number (Jovanović et al. 2010). To determine PCV, the cell suspension was first mixed vigorously, and aliquots of 10 ml poured into 15-ml Falcon tubes. The tubes were kept in an upright position at 4 °C for 3 days to allow the cells to fully settle down. Afterwards, the PCV was read out using the scale of the Falcon tube and recorded as percentage of the total volume used.

### Subcellular characterisation

Subcellular compartments of *Catharanthus* cells were visualised as to study intracellular differences between the two cell strains. Actin filaments, Golgi vesicles, peroxisomes, and tonoplast were labelled with fluorescent markers by transient transformation of the two *Catharanthus* cell strains using particle bombardment. The constructs targeting the respective subcellular structures are listed in Supplementary Table S1. Cells at the onset of the proliferation phase, at day 3 after sub-cultivation, were transformed as described previously (Maisch et al. 2009). The transformed cells were allowed to express the respective marker for an additional 24 h before analysis under an AxioImager Z.1 microscope (Zeiss, Jena, Germany) equipped with an ApoTome microscope slider for optical sectioning and a cooled digital CCD camera (AxioCam MRm; Zeiss). The YFP and GFP signals were observed through the filter sets 46 HE (excitation 500 nm, beam splitter: 515 nm, emission: 535 nm, Zeiss), and 38 HE (excitation 470 nm, beam splitter: 495 nm, emission: 525 nm, Zeiss), respectively.

Mitochondria were visualised with 100 nM MitoTracker Red FM, a red-fluorescent dye. The cells were observed immediately without incubation or washing (Agnello et al. 2008). To get high-resolution images, an AxioObserver Z1 inverted microscope (Zeiss, Jena, Germany) was used, equipped with a laser dual spinning disc device from Yokogawa (Yokogawa CSU-X1 Spinning Disc Unit, Yokogawa Electric Corporation, Tokyo, Japan), and a cooled digital CCD camera (AxioCam MRm; Zeiss). Images were recorded using the 561-nm emission line of the Ar-Kr laser and a Plan-Apochromat 63 × /1.44 DIC oil objective operated via the Zen 2012 (Blue edition, Zeiss) software. We conducted quantitative image analysis using ImageJ (https://imagej.nih.gov/ij/) to measure mitochondrial shape and density. In brief, for the shape measurements, mitochondrial images were first converted into binary images. Then, the mitochondrial structures were fitted with ellipses to allow measurement of the length of these structures and automatically selected using the “Analyse particle” tool of the Image J software. Specificity was reached by adjusting the selection size to 1–500 pixels and allowing circularity for the full range (0–1), which allowed quantifying the punctate as well as the filamentous mitochondrial structures. In case of quantifying mitochondrial density, the images from the ZEN software were first converted into the 16-bit format and later translated into binary images. Once again, the “Analyse particle” tool of the ImageJ software was then used to quantify mitochondria, setting “size” parameters adjusted to “10 infinity” and the “circularity filter” was set to “0–1”. The total area of the mitochondrial structures within the respective cell was then related to the total cross area, to yield a value for mitochondrial coverage, which was then followed by both the cell lines over the culture cycle of 15 days. Three biological experimental replicates were analysed, and the measurements were estimated for ≥ 1300 cells. A similar procedure of quantitative image analysis was employed using ImageJ to measure peroxisomal density in both the transformed *Catharanthus* cell strains containing the chimeric construct targeting peroxisomes.

### Estimation of sucrose dependency

If not stated otherwise, both cell strains were generally cultivated in fresh and autoclaved growth medium containing sucrose (30 g/l) as a major source of carbon. However, in order to establish the effect of substrate limitation, both cell strains were cultivated in growth medium containing various concentrations of sucrose (15–100 g/l) along with Gamborg B5 salts (3.21 g/l) and 2,4-D (5 mM), with pH adjusted to 5.6 ± 0.03. Potential effects on cell viability due to these sucrose concentrations were followed over a period of 6 days. To monitor sugar consumption from the medium in a minimal invasive manner, a portable Brix refractometer (Model PAL, Atago Co. Ltd., Tokyo, Japan) was used. Small aliquots of the vacuum filtered medium were collected under sterile conditions and then used to determine the refractive index by measuring the rotation of polarised light due to sugar chirality. The refractive index allows then to determine the sugar concentration. Twenty microlitres of vacuum-filtered medium without the cells was applied on to the refractometer and the Brix values were documented for both the cell strains along the 15-day cultivation period. To study the effect of different sucrose concentrations on alkaloid accumulation of these cell strains, cells from both strains were transferred, at day 7 of sub-cultivation, into fresh medium without auxin (2,4-D), but complemented with different sucrose concentrations. The cells were allowed to grow over a period of additional 6 days during which alkaloid content and cell volume were monitored.

### Elicitation and precursor feeding

To probe for elicitation by jasmonates, stationary cells (day 7 after sub-cultivation) were transferred into medium without 2,4-D to retain the cells in the stationary phase and support accumulation of secondary metabolites. Jasmonic acid (JA) and methyl jasmonate (MeJA) were added at 100 μM. Since MeJA is a volatile compound, special care was taken to wrap the filter caps of the flasks with tissue paper and aluminium foil. In a different set of experiments, it was attempted to further boost the production of downstream alkaloids by feeding upstream precursors from the vinca alkaloid pathway, such as tabersonine (1.2, 2.4, 3.6 μM), catharanthine (1.2 μM), or vindoline (0.88, 1.6, 2.4 μM). This precursor feeding was also combined with elicitation by MeJA in some experiments, to study the effects of such multiple triggers, which could favour the activity of such a complex biosynthetic pathway. Jasmonic acid and methyl jasmonate were dissolved in ethanol (EtOH); the pathway precursors (tabersonine, catharanthine, and vindoline) were dissolved in methanol (MetOH). Hence, solvent controls were also included, where the cells were treated with the corresponding concentrations of EtOH (0.04%) or MetOH (0.04%) alone, as mock controls. For both elicitation and precursor feeding, the cells were further monitored for up to additional 10 days following the treatment. The cells and their respective culture medium were collected and frozen at − 20 °C before lyophilisation for 3 days. The lyophilisates were then used for further analysis.

### Extraction of vinca alkaloids

Vacuum filtration technique was used to collect cells and medium exclusively at different experimental time points for both C1 and C4 strains. For alkaloid extraction, approximately 2 g of filtered cell material and all of the culture filtrate per experimental flask were collected exclusively and frozen at − 20 °C and further lyophilised for ~ 72 h. The lyophilised cell material and the filtrate were suspended in ~ 1 to 2 ml of MetOH, respectively. These extracts were further lysed by ultrasonication for 2 min (amplitude 100%, 0.5-s pulse) using a high-efficiency ultrasound device (UP 100H, Hielscher Ultrasonics GmbH, Teltow, Germany). Extracts were spun down for 10 min with 10,000* g* at 25 °C. The supernatant containing the alkaloids was filtered with a 0.45-μm needle type Chromafil PET-20/15 MS filter (Macherey–Nagel GmbH & Co. KG, Düren, Germany) into the autosampler vials (WIC4200, WICOM Germany GmbH, Heppenheim, Germany). Individual stock solutions of the alkaloid standards such as catharanthine, tabersonine, vindoline, vinblastine, and vincristine were prepared at a concentration of 1 mg/ml in MetOH. These stock solutions and the alkaloid extracts were stored at − 20 °C for further analysis.

### Qualitative metabolite analysis by liquid chromatography-mass spectrometry (HPLC–DAD-ESI–MS/MS)

For sensitive detection of vinca alkaloids, a LXQ Linear Ion Trap MSn system (Thermo Fisher Scientific, Waltham, MA, USA) equipped with a Finnigan Surveyor HPLC–PDA was used. The extracts were separated on a Phenomenex Luna C18 column (4.6 mm × 250 mm, 5 μM particle size) with a gradient of 10 mM ammonium acetate, pH 6.0 (solvent A), and LC-grade MetOH (solvent B) as mobile phase. Flow rate was set to 500 μl/min. The eluent profile (% of solvent A/ % of solvent B) was as follows: 0–5 min, linear gradient from 30:70 to 10:90, and 5–23 min, gradient elution from 10:90 to 30:70. Masses were detected using an ion trap mass spectrometer coupled with electrospray ionisation and operating in a positive mode. Spray voltage was set at 4 kV, capillary voltage at 33 V, capillary temperature 350 °C, and the tube lens voltage to 70 V. The full mass scan covered the range from m/z 100 to 1000. Retention times, pseudo-molecular ions [M + H]^+^, and MS^2^ fragment ions for the analysed alkaloids are given in Supplementary Table S2. HPLC–ESI–MS/MS chromatograms for each of the standard compounds used in our study are shown in Supplementary Fig. S1.

### Quantitative metabolite analysis by high-performance liquid chromatography (HPLC–DAD)

Chromatographic separation was carried out on an Agilent Eclipse XDB-C18 column (4.6 mm × 250 mm, 5 μm particle size). The chromatographic system was an Agilent-1200-Series HPLC system, consisting of a G1322A degasser, a G1311A quaternary pump, equipped with a G1329A auto sampler, and a G1315D diode array detector (Agilent Technologies, Santa Clara, USA) coupled with the Agilent ChemStation software. The mobile phase consisted of 45 parts of acetonitrile, 15 parts of methanol, and 40 parts of ammonium acetate (25 mM), supplemented with 0.1% triethylamine at a flow rate of 1 ml/min for 30 min (Siddiqui et al. 2011). The injection volume was 30 μl. Alkaloids were identified by comparison of the UV spectra at 297 nm, and retention time with those of authentic standards. They were quantified using the calibration curves of the standards. Standards and their retention times are listed in Supplementary Table S3.

### RNA extraction and semi-quantitative RT-PCR

Vacuum-filtered cell material was collected from both cell strains. Approximately 100 mg of filtered cell material was transferred to a 2.0-ml reaction tube, frozen in liquid nitrogen, and kept at − 80 °C until RNA extraction. These frozen cells were then ground into a fine powder using quartz sand and pestle. Total RNA was extracted using the innuPREP Plant RNA Kit (Analytik Jena, Jena, Germany), a column-based extraction method, following the manufacturer’s protocol. The RNA was DNase (New England Biolabs, Ipswich, USA) treated, to remove any contaminating genomic DNA. The cDNA was synthesised from 2 μg of total RNA template, and the subsequent PCRs were performed using the Protoscript First strand cDNA synthesis kit and *Taq* DNA polymerase (New England Biolabs, Ipswich, USA) following the manufacturer’s instruction. One 20 μl reaction mixture comprised 1 μl of a 10 mM dNTPs mix, 0.5 μl Taq-polymerase, 2 μl of 10 X Taq-buffer, 1 μl of reverse and forward primers (5 pmol each), 1 μl of cDNA, and the corresponding amount of DNAse-free water. The RT-PCR reactions were carried out as per the following cycling thermal parameters: initial denaturation at 95 °C for 3 min followed by 30 cycles of denaturation at 95 °C for 30 s, primer annealing at 50–55 °C, depending on the respective primer pairs (Supplementary Table S4) for 30 s, and extension at 68 °C for 30 s. This was followed by a final extension of 68 °C for 5 min. Expressed protein (*exp*) was used as a reference gene for this study due to its high expression stability (Pollier et al. 2014). Primers were designed with the Primer3 software (http://primer3.ut.ee/) and synthesised by Sigma-Aldrich (Munich, Germany). The complete list of primers and accession numbers used in our study are presented in Supplementary Table S4.

## Results

### The strains C1 and C4 differ with respect to morphology and growth pattern

Plant cells in suspension exhibit morphological and physiological heterogeneity, limiting their use for producing secondary metabolites (Dougall 1987). For *Catharanthus roseus* as well, suspension cell strains generated from single seedlings had shown unstable production of vinca alkaloids, indicative of metabolic heterogeneity (Deus-Neumann and Zenk 1984). As to design a strategy to study and/or improve the metabolic potential of these *Catharanthus* cell strains, it was imperative to first characterise them morphologically.

**Fig. 2:**
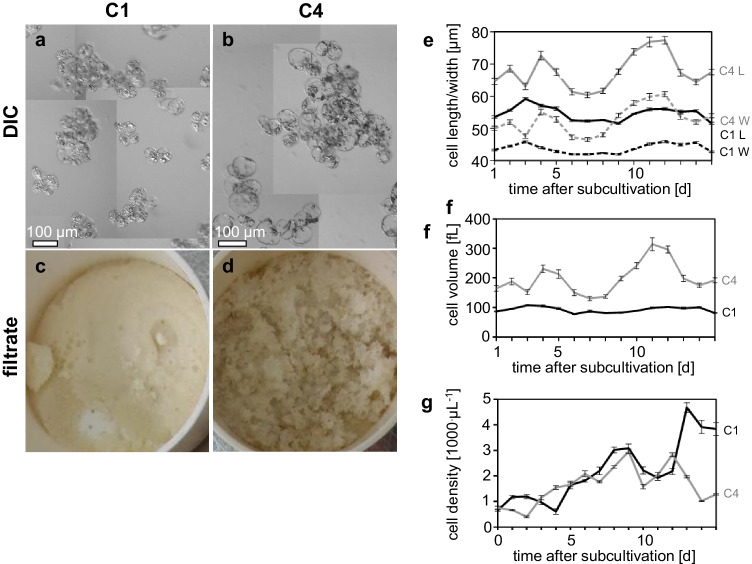
Comparative phenotypic characterisation of the C1 and C4 cell strains. **a, b** Representative images of cell aggregates in the differential interference contrast. **c, d** Macroscopic appearance of culture filtrate. **e–g** Change of cellular parameters over the culture cycle. **(e)** Cellular dimensions, L cell length (solid strains), W cell width (dashed strains). **f** Cell volumes inferred from the cellular dimensions assuming a spherical shape. **g** Cell density. Black curves C1, grey strains C4. Data show mean ± standard error, *n* ≥ 500, and 5 biological replicates

As a prerequisite, the behaviour of the cultures had to be calibrated for stability. This was successful since growth indices (ratio of fresh weight at the end of the culture cycle over that of the initial inoculum) were constant over more than 20 cycles (Supplementary Figure S2a). However, the growth indices of C4 fluctuated more widely than those of C1. But even in C4, these fluctuations remained < 20% of the average level. The higher growth index of the C4 strain meant that any attempts towards establishing co-cultivated cultures of the C1 and C4 strains together in the same flask were unsuccessful. While establishing the growth stability in the cultures of co-cultivated strains, we observed that the C4 strain dominates the co-cultivated cultures due to higher growth index. Thus, all experimental measurements were performed exclusively on these two strains. We also observed both cell strains to form aggregates in liquid culture, although the aggregates of C4 were much larger (Fig. 2b) than those of C1 (Fig. 2a), both in terms of cell number and cell size. The aggregates of C1 exhibited a smooth and fine structure (Fig. 2c), whereas C4 was friable (Fig. 2d). These differences were also reflected on the level of cell size (Fig. 2e). The cells of strain C4 were significantly larger, both in length and in width, which on the level of estimated cell volume made up a factor of 1.6 times compared to strain C1 (Fig. 2f).

From the time course of cell density (Fig. 2g), the average doubling time could be inferred for both cell strains using an exponential growth model. Here, C4 duplicated slightly faster with 4.2 days as compared to C1 with 4.8 days. However, this proliferation was more persistent in C1 beyond 7 days (the usual time of sub-cultivation), while it was discontinued after day 7 in C4. As a result, C4 had increased in cell density fourfold after 15 days, while density had increased sevenfold in the case of C1. The peak density for C4 was reached already on day 9, while for C1 the peak was reached only on day 13 (Fig. 2g). This time course of cell density was also mirrored on the level of fresh and dry weight, and packed cell volume (Supplementary Figure S2). The fresh weight showed an exponential curve for C4 until day 8, followed by a plateau phase. However, in C1, fresh weight increased more slowly, at an almost constant rate, with only small increases observed between day 5 and day 6 and, again, between day 12 and day 13 (Supplementary Fig. S2b). The increase of fresh weight was strongly correlated with the increase in cell number. In contrast, the trend was reversed, if dry weight was considered (consistent with the finding that cells of C4 were larger (Fig. 2f)). Here, the values of C1 were much higher than those of C4 over the entire measurement period (Supplementary Fig. S2c). If packed cell volume as global readout was analysed, the increase over time was nearly linear for C1 (Supplementary Fig. S2d), while there was a pronounced sigmoidal time course for C4 with a lag phase prior to day 4, an exponential increase between days 4 and 7, and saturation from day 7. Thus, the two strains not only differ with respect to cellular morphology but also with respect to their growth patterns.

### Cells of strain C4 are more vacuolated and show mitochondrial fusions

**Fig. 3:**
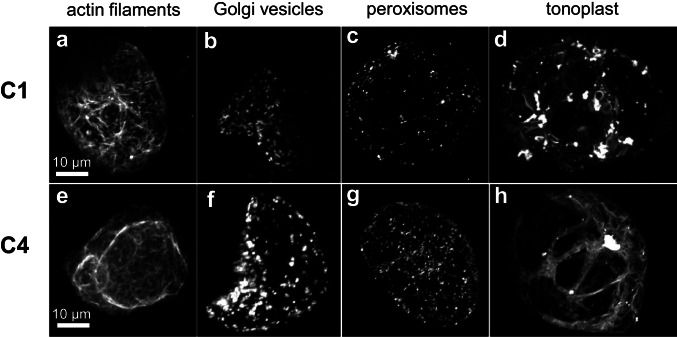
Visualisation of subcellular compartments in the C1 and C4 cell strains. Representative geometric projections of z-stacks collected by spinning-disc confocal microscopy after transient transformation of the two *Catharanthus* cell strains with GFP-tagged markers. **a, e** FABD2-GFP**–**labelled actin filaments, **b, f** ST-GFP**–**labelled Golgi vesicles, **c, g** POX-YFP**–**labelled peroxisomes, and **d, h** NtTPC1A-GFP**–**labelled tonoplast

Alkaloid biosynthesis partitions to different sub-cellular compartments, including cytoplasm, vacuole, tonoplast membrane, and endoplasmic reticulum, are directly involved in alkaloid biosynthesis. The enzymes of the pathway are strictly compartmentalised, which requires pathway intermediates to be transported from one compartment to the next (Facchini 2001; Mahroug et al. 2007; Guirimand et al. 2011). Since the two cell strains, C1 and C4, differed in morphology, they might also differ with respect to their subcellular compartmentalisation. To get insight into their subcellular architecture, we transformed cells transiently with different fluorescent markers labelling actin filaments, Golgi vesicles, peroxisomes, and tonoplast, respectively (Fig. 3a–h). The most salient differences detected between the two cell strains concerned the actin cytoskeleton (Fig. 3a, e) and the tonoplast (Fig. 3d, h). While strain C1 displayed a finer meshwork of cortical actin, in C4 actin was forming prominent transvacuolar actin cables. Conversely, the tonoplast was more subdivided into smaller lacunae in C1, while in C4, the central vacuole was more prominent, filling most of the cell interior. In addition, peroxisomes appeared to be more abundant in C4 as compared to C1, with almost tenfold higher amounts observed in C4 strains (Fig. 3c, g and Supplementary Fig. S3).

**Fig. 4:**
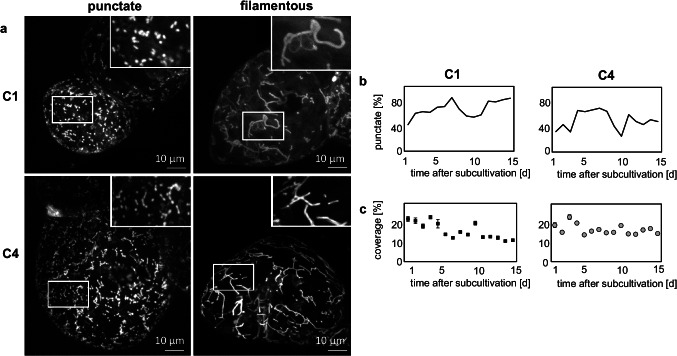
Qualitative and quantitative analysis of mitochondria in the C1 and C4 cell strains. **a** Representative images collected by spinning-disc confocal microscopy making use of the fluorescent signal from MitoTracker Red FM. Punctate and filamentous mitochondria were observed. Inset shows a zoom-in of the region marked by the white frame. **b** Frequency of cells with punctate mitochondria over the culture cycle. **c** Total mitochondrial coverage over the culture cycle. Data show mean ± SE, *n* ≥ 1300 cells

Since alkaloid biosynthesis is an energy-intensive process, mitochondria were studied in more detail. In both cell strains, mitochondria displayed different forms, ranging from punctate to filamentous, mesh-like structures (Fig. 4a). This variation of mitochondrial shape was then followed over a period of 15 days for both cell strains (Fig. 4b). Here, the punctate pattern was found to be dominant in C1, while in C4, the frequency of punctate mitochondria was significantly lower (Fig. 4b). There was an undulating rise and fall in the punctate pattern in both cell strains. Punctate mitochondria became more frequent until day 7, followed by a decline until day 10, whereupon their incidence grew again. In summary, while the overall pattern was parallel in both *Catharanthus* cell strains, the amplitude for the frequency of punctate mitochondria was higher in C1 as compared to that in C4. In contrast to mitochondrial shape, total mitochondrial coverage behaved in a similar way for both strains, being higher in the beginning of the culture cycle (Fig. 4c) and then decreasing slowly. Thus, the two strains do not differ so much in mitochondrial number, but rather in the partitioning between different mitochondrial states. While mitochondria in strain C1 are mainly small, they tend to expand and fuse in strain C4.

### Sugar consumption rate is higher in strain C1

Disaccharides in the culture medium are an important source of energy to support cell growth, and a source of carbon for generating the carbon-rich alkaloids as well. In a range between 15 and 60 g/l, viability was close to 100% (Supplementary Fig. S4a). When the sucrose concentration exceeded 60 g/l, viability dropped progressively. At 100 g/l sucrose, cell viability in C4 was about 75% over the 6-day growth period. Sugar consumption rates were also quantified for both cell strains by following the sugar content in the medium over time using a Brix refractometer (Supplementary Fig. S4b). For strain C1, sugar concentration showed a sharp decline after just 3 days. Already on day 6, the sugar content had decreased to a barely detectable level. In contrast, the sugar content in strain C4 started to decline from day 4, which then continued less rapidly compared to C1 until day 8, after which it stabilised at a value around 0.5%. Thus, the rate of sugar consumption is higher in C1 as compared to C4.

## Strain C1 shows a strong expression of *peroxidase 1*

**Fig. 5:**
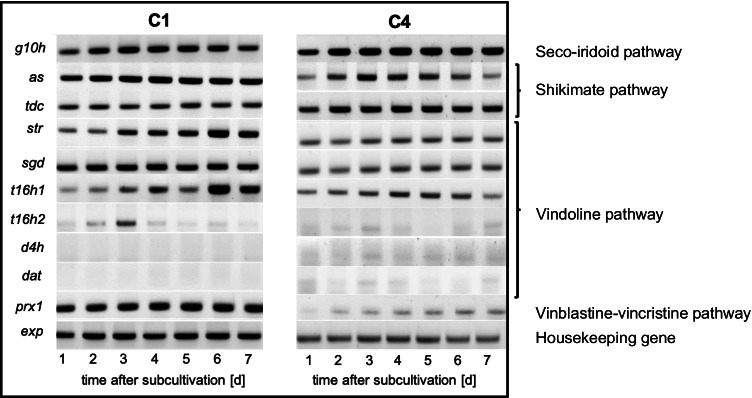
Expression profile of TIA biosynthesis pathway genes in C1 and C4 cell strains. Time course of transcripts participating in TIA biosynthesis. Representative RT-PCR profiles are shown for both *Catharanthus* cell strains. Seco-iridoid pathway represented by *geraniol 10-hydroxylase* (*g10h*); shikimate pathway by *anthranilate synthase* (*as*), *tryptophan decarboxylase* (*tdc*); TIA intermediates production represented by *strictosidine synthase* (*str*), *strictosidine β-D-glucosidase* (*sgd*); vindoline pathway by *tabersonine 16-hydrolxylase 1 and 2* (*t16h1* and *t16h2*), *deacetoxyvindoline 4-hydroxylase* (*d4h*), *deacetylvindoline 4-O-acetyltransferase* (*dat*); vinblastine-vincristine pathway by *peroxidase 1* (*prx1*), and e*xp* (*expressed protein*) served as a reference housekeeping gene

Expression of several key biosynthesis genes for the various biosynthetic branches (seco-iridoid pathway, shikimate pathway, vindoline pathway, and vinblastine-vincristine pathway) involved in the synthesis of vinca alkaloids was analysed exclusively in these two cell strains using semi-quantitative reverse transcriptase PCR (Fig. 5). We observed that geraniol 10-hydroxylase (*g10h*), as key enzyme of the seco-iridoid pathway, was expressed strongly from between days 2 and 6 in C1, while in C4 it remained high even at day 7. Anthranilate synthase (*as*), as initial step of the indole pathway, was strongly and constitutively expressed in C1, while its expression was weaker in C4 and remained transient (days 3–5). Tryptophan decarboxylase (*tdc*), which converts tryptophan into tryptamine, was constitutively expressed in both the cell strains with a stronger expression in C4. Strictosidine synthase (*str*) and strictosidine β-D-glucosidase (*sgd*), which are involved in preparing the precursors for the TIA biosynthesis pathway, were constitutively expressed in C4, albeit to lower levels. In C1, *sgd* was stable at a higher level, while *str* increased significantly during the second half of the culture cycle. The expression of genes within the vindoline biosynthesis pathway exhibited even more profound differences between the two cell strains: Tabersonine 16-hydroxylase 1 (*t16h1*) showed expression in both cell strains, however with an upregulation in C1. The expression of tabersonine 16-hydroxylase 2 (*t16h2*) was weak in cell line C1, with a stronger expression at day 3, while a very weak expression could be detected in C4 at days 2, 3, and 7. For the genes involved in the final steps of the vindoline biosynthesis pathway, namely deacetoxyvindoline 4-hydoxylase (*d4h*) and deacetylvindoline 4-O-acetyltransferase (*dat*), no expression could be detected in C1. In C4, a very weak signal was found for *d4h* on days 3, 5, 6, and 7, and a comparably weak signal for *dat* on days 3, 4, and 7. The peroxidase gene (*prx1*), which is proposed to be involved in the final step of the production of the bisindole alkaloid vinblastine from anhydrovinblastine, was strongly expressed in strain C1 throughout the growth period but was much lower in strain C4. This difference in the amplitude of *prx1* transcripts was the most significant among the tested genes.

### Auxin depletion promotes the accumulation of catharanthine in C4

**Fig. 6:**
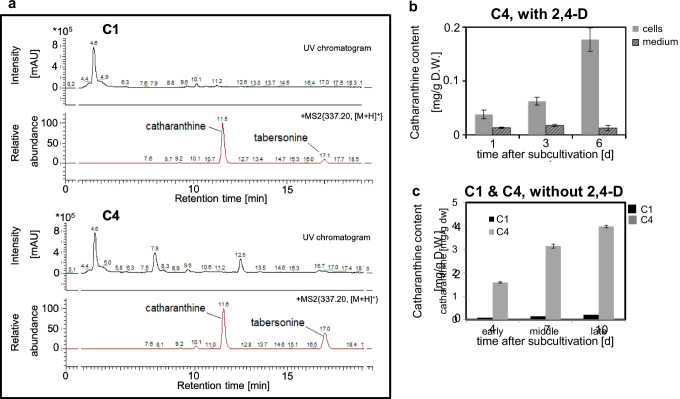
Qualitative and quantitative profiling of the alkaloids in C1 and C4 cell strains. **a** Representative HPLC–DAD-ESI–MS/MS chromatograms from the alkaloid extracts of the two *Catharanthus* strains. UV detection was carried out at 210 nm, and MS^2^ was performed on *m/z* 337 ([M + H]^+^ of catharanthine and tabersonine). **b** Time course for the accumulation of intracellular and secreted catharanthine in C4 cells in the presence of standard medium containing 2,4-D as exogenous auxin. **c** Stimulation of catharanthine accumulation by auxin depletion (medium without 2,4-D) in strains C1 and C4 during different periods of the culture cycle. Data in **b** and **c** show mean ± SE from 3 independent experimental series

Both *Catharanthus* cell strains, C1 and C4, were analysed, qualitatively as well as quantitatively, for the levels of the alkaloids they accumulated. During the growth phase, both strains were cultivated in the growth medium in presence of the artificial auxin 2,4-D to support biomass accumulation. As a more sensitive analytical approach, LC–MS technology was employed to qualitatively assess the profile of alkaloids. Catharanthine and tabersonine could be detected in the cell material of both cell strains (Fig. 6a). Vindoline and the other high-value alkaloids vinblastine and vincristine could not be detected in any of the strains during the growth phase. These growth phase samples were then also subjected to quantitative analysis using a less sensitive HPLC–DAD platform. Neither catharanthine nor tabersonine could be quantifiably detected in both the cell material and medium of C1 cells. However, in C4 strains, catharanthine was detected in small amounts in both its cell material and medium (Fig. 6b). Intracellular catharanthine accumulated significantly over time; however, the amount of catharanthine recovered from the filtered medium was much lower. These findings were congruent with our LC–MS results, indicating that the alkaloids accumulated, however only to very low abundance during growth phase, irrespective of the cell strain.

In the next step, the alkaloid profile was determined in response to auxin depletion, established by cultivation medium without 2,4-D. Since auxin is essential for cell division, thus, both strains were in stationary phase which should promote the synthesis of secondary metabolites. During pilot studies, both catharanthine and tabersonine could be detected in both cell strains, with catharanthine content increasing over time. Therefore, cells were sampled early (at day 4), in the middle (at day 7), and late (at day 10) during this stationary phase caused by auxin depletion. Once again, catharanthine was the only alkaloid that could be quantitatively detected in both strains (Fig. 6c). In addition, strain C4 accumulated substantially more catharanthine over time as compared to the growth phase. While the levels were just under 0.2 mg/g in the auxin-supplemented medium on day 6, they had increased around 20-fold to ~ 4 mg/g on day 10 of the stationary phase.

### Catharanthine accumulation can be elicited in strain C4 by exogenous jasmonates

Since auxin depletion was successful in promoting alkaloid accumulation, we further studied the effect of chemical elicitation. No matter, whether 2,4-D was present (growth phase) or omitted (stationary phase), viability for both cell strains was maintained at a very high level during growth phase level (Supplementary Fig. S5a, b). During the stationary phase, the viability of C4 decreased slightly to 93% on day 6, whereas for C1 there was no change. The additional closing of the filter caps of the flasks which disrupted oxygen access to the cells had a slight negative effect on the viability of C1, whereas it showed a comparatively more pronounced effect on the viability of C4. In case of stationary cells, the 0.04% EtOH (control solvent for phytohormones) significantly reduced the viability of C1 from 97% on day 1 to 89% on day 6, while C4 displayed less sensitivity to EtOH (dropping only from 94 to 90% over the same period). The effect of 0.04% and 0.08% MetOH (the solvent controls for the precursor feeding experiment) on C4 is also lower than the effect of EtOH, even at higher concentrations. In case of MeJA treatment (which included combination effects of EtOH, closed caps, and MeJA), C1 showed a reduction in viability from 96 to 80%, whereas C4 remained quite stable with a decrease from 95 to 90%. In summary, the viability of C4 was at a slightly lower level for untreated samples, but remained more stable than C1, when subjected to various treatments indicating better cellular homeostasis for C4 cells. To test whether sugar would be limiting, we measured catharanthine accumulation over higher sucrose concentrations (Supplementary Fig. S6a). In fact, catharanthine accumulation could be stimulated in both lines, albeit to a different extent and only up to 60 g/l of sucrose. For higher concentrations, the abundance of catharanthine dropped again.

**Fig. 7:**
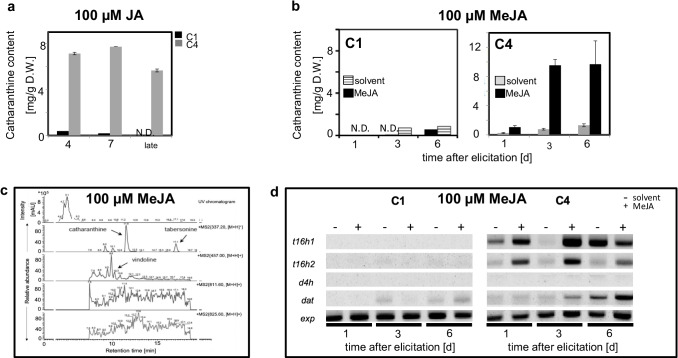
Effect of jasmonate elicitation on alkaloid accumulation in the C1 and C4 cell strains. **a,b** Time course of catharanthine accumulation in response to elicitation with JA (100 μM, **a**) or MeJA (100 μM, **b**) in strains C1 and C4 under conditions of auxin depletion (no 2,4-D). Solvent control in **b** consisted 0.04% EtOH. Data show mean ± SE from 3 independent experimental series. N.D. means that catharanthine was not detectable. **c** Representative HPLC–DAD-ESI–MS/MS chromatograms of extracts from auxin-depleted, JA-elicited C4 cells; *m/z 337* (catharanthine and tabersonine), *m/z 457* (vindoline), *m/z 811* (vinblastine), *m/z 825* (vincristine). **d** Representative gels showing amplicons from RT-PCR for genes representing the vindoline biosynthesis pathway. Time courses of expression were measured for both strains in response either to the solvent (0.04% EtOH) as a mock control, or to 100 μM MeJA as elicitor. *t16h1* (*tabersonine 16-hydrolxylase 1*), *t16h2* (*tabersonine 16-hydrolxylase 2*), *d4h* (*deacetoxyvindoline 4-hydroxylase*), *dat* (*deacetylvindoline 4-O-acetyltransferase*), and exp (*expressed protein*) served as a reference housekeeping gene

Since alkaloid accumulation, in the biological context, represents a strategy against herbivory, a stress that is conveyed by activation of jasmonate signalling, we used jasmonic acid (JA) as an elicitor. This stimulated a clear accumulation of catharanthine in strain C4, but not in strain C1 (Fig. 7a). The effect in C4 was most prominent at day 4 with a factor of 4.5 over the catharanthine levels in the control without JA (Fig. 6c). However, this surplus decreased with time because catharanthine also accumulated over time in the auxin-depleted but non-elicited condition (Fig. 6c). Still, at day 7, the addition of JA had more than doubled the catharanthine content compared to the non-elicited control. Even on day 10, an increase of around 50% over the control was seen. The highest abundance of catharanthine, 7.7 mg/g dry weight, was found on day 7 of JA-elicited, stationary C4 cells. For C1, only very low values of catharanthine were detected, which further decreased steadily along the course of the JA treatment.

Since the access of JA to the relatively compact cell clusters might be limiting, we tested for the volatile derivative MeJA, sampling on days 1, 3, and 6 after the treatment, and using the more sensitive LC–MS analysis. Now, in addition to catharanthine, also tabersonine could be detected. Interestingly, minute traces of vindoline were observed in some of the samples (Fig. 7c). However, catharanthine remained the only tangible alkaloid, accumulating to levels that could be quantified, also in these MeJA-treated cell strains (Fig. 7b). While the content in C1 increased only to minute levels, C4 was more responsive. Here, from day 3, we observed a significant increase of catharanthine in the C4 strain to ~ 10–11 mg/g dry weight on day 6, which was much higher than the values seen for JA elicitation (Fig. 7a). Vinblastine and vincristine were not detected in any of the strains, irrespective of whether JA or MeJA was used for elicitation.

To get more insight into these strain differences in the accumulation of alkaloids, we examined the expression of the vindoline pathway genes under these conditions (Fig. 7d). In C1, neither *t16h1* nor *t16h2* was expressed, despite MeJA elicitation (which did cause a slight expression of *dat* at day 6). In contrast, in C4, there was a clear induction of *t16h1* and *t16h2* as early as 1 day after elicitation, and from day 3, also a significant increase in steady-state transcript levels of *dat* in C4. Thus, several genes required for the conversion of tabersonine into vindoline became activated in strain C4.

### Feeding alkaloid pathway precursors to strain C4 leads to vincristine

**Fig. 8:**
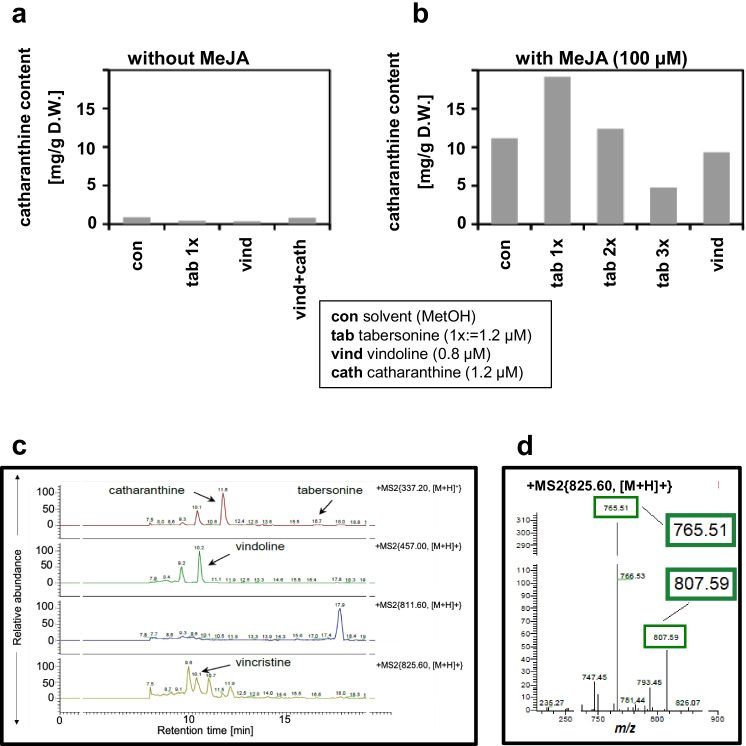
Alkaloid profiles in strain C4 in response to precursor feeding and elicitation with MeJA. **a,b** Intracellular catharanthine accumulation in the absence **a** or after elicitation with **b** 100 μM MeJA after feeding different precursors. **c** HPLC–DAD-ESI–MS/MS chromatograms of intracellular alkaloids extracted from C4 strain treated with a combination of 100 μM of MeJA and 0.8 μM of vindoline over a period of 6 days. Various extracted ion chromatograms are shown in different colours specific to different alkaloids. Various extracted ion chromatograms in different colours specific to different alkaloids. **d** MS^2^ spectrum of *m/z* 825 ([M + H]^+^ of vincristine) from cell extracts described above **c**

The above results gave clear indications that MeJA elicitation was able to elicit catharanthine production in C4 cells (but not in C1) (Fig. 7b). Moreover, some of the transcripts needed for the conversion of tabersonine into vindoline were induced as well (Fig. 7d). Still, only trace amounts of vindoline became detectable. Thus, there seems to be a bottleneck, here. We wondered whether the vindoline precursor, tabersonine, might be limiting, and whether we would be able to remove this bottleneck, by feeding tabersonine (1.2 μM). We asked further whether feeding of the downstream product of this limiting metabolic branch, vindoline (0.8 μM), might lead to the accumulation of vinblastine or vincristine. Since these compounds emerge from the fusion of the catharanthine and the vindoline moieties (Fig. 1), we conducted these feeding experiments also in a variant, where catharanthine (1.2 μM) was added as well and allowed accumulation over a period of 6 days (Fig. 8a). To ensure that metabolic competence was fully unfolded, the cells were again elicited by MeJA (Fig. 8b).

In fact, in the absence of MeJA, none of the tested precursors had any discernible effect on catharanthine biosynthesis (Fig. 8a). In combination with MeJA, 1.2 μM tabersonine stimulated the accumulation of catharanthine, but only for small amounts of tabersonine (Fig. 8b). When the concentration of tabersonine was doubled or tripled, the catharanthine levels decreased; for 3.6 μM of tabersonine, they dropped even below the level seen without tabersonine, indicative of channelling the common precursors (stemmadenine) towards the tabersonine-vindoline branch of the pathway, if tabersonine levels were high. In contrast, feeding of vindoline (0.8 μM), the downstream product of the tabersonine branch, was not effective in changing catharanthine accumulation. When we probed with LC–MS, we found for the combination treatment of MeJA and vindoline, in addition to tabersonine and catharanthine, small amounts of vincristine (Fig. 8c). The identity was verified by the presence of the qualifier fragment ions, 765.51 and 807.59, which were confirmed by the standard compound (Fig. 8d). Encouraged by this finding, we then tried to promote vincristine accumulation by a combination of MeJA and vindoline (3 different concentrations—0.88, 1.6, 2.4 μM) on C4 in several replications. Unfortunately, although the qualifier fragment ions (765.51 and 807.59) for vincristine were found in these treated samples for the peak of a compound at an appropriate retention time, they were superimposed by other masses, which suggested extremely low amounts, or an extremely unstable production (data not shown). Vinblastine was not detected in any of the elicited treatments. However, we observed an unknown peak at a retention time of 17.9 min with m/z 811.60 (specific for vinblastine). However, the expected retention time of vinblastine was quite different (10.2 min). Thus, the identity of this compound remains unresolved.

## Discussion

In the current work, we showed that two cell strains of *Catharanthus roseus* differ in their metabolic competence. By tailoring elicitation and by feeding limiting precursors, we could modulate metabolic flows and show that one of these strains can produce trace amounts of vincristine. These findings stimulate different questions: What are the branching points that determine this metabolic competence and is it possible to modulate those? Are there morphological or cellular markers of this differential metabolic competence? How does the finding of differential metabolic competence bear on biotechnological applications based on plant cell fermentation?

### The vindoline bottleneck: a crucial factor for alkaloid accumulation

Vindoline biosynthesis is very complex (Fig. 1) and involves transformation of the precursor tabersonine by seven enzymatic reactions encompassing multiple tissues (Qu et al. 2015). Vindoline and catharanthine are then further combined to produce anhydrovinblastine which is then converted to vinblastine and vincristine (Liu et al. 2016). While tabersonine and catharanthine were detected in both strains, vindoline could not be detected in either of these cell strains without elicitation, although it was found in C4, when they were elicited with MeJA (Fig. 7c). Even though C1 strains were more stable in their growth, inability to produce vindoline even upon elicitation seems to be the major metabolic difference between these two strains. Thus, in general with regard to these cell strains, vindoline which is crucial for synthesising the high-value vinca alkaloids seems to be the limiting factor, consistent with the fact that it is not on the list of alkaloids that have been detected in suspension cultures (for a comprehensive review, see van Der Heijden et al. 1989). Compared to catharanthine, vindoline biosynthesis is more tightly controlled by developmental and environmental conditions (St-Pierre et al. 1998). Its complex biosynthetic pathway involves intermediates produced specifically in epidermal as well as mesophyll cells (Levac et al. 2008; Besseau et al. 2013). Metabolic engineering of the vindoline pathway is very challenging as well, as there are many competing branches and these shunt products are formed by the same enzymes involved in the production of vindoline (Kellner et al. 2015; Edge et al. 2018). Recently, Sun et al. (2017) managed to shift tabersonine from competing pathways to the vindoline pathway by overexpressing *t16h* and *16omt*, the genes for the first two enzymes in the vindoline pathway, in hairy root cultures. But in consequence they also detected new metabolites, indicating that this shift also activated other biosynthesis pathways, branching off from the main route towards vindoline. Our data show that at least one of the *t16h* transcripts is expressed in both strains (Fig. 5), while transcripts for deacetoxyvindoline 4-hydroxylase (d4h) and deacetylvindoline 4-O-acetyltransferase (dat), the final two steps upstream of vindoline, are missing, which might be the real bottleneck behind the lack of vindoline. Therefore, while heterologous expression of the vindoline pathway genes in yeast (Qu et al. 2015; Liu et al. 2021) has been successful, vindoline has remained a major bottleneck in the production of downstream bisindole alkaloids in *Catharanthus* cell strains.

### Jasmonates preferentially increase fluxes through the catharanthine branch than the vindoline branch

Jasmonates are important constituents of stress signalling pathways in plants and upregulate expression of defence genes encoding toxic secondary metabolites, so-called phytoalexins (Montiel et al. 2011). For this reason, jasmonates are commonly used to elicit secondary compounds in cell culture, for instance alkaloids, such as nicotine (Rajabi et al. 2017). This works as well to induce TIA production in *Catharanthus* cells through a massive network of jasmonate-responsive transcription factors that modulate TIA biosynthesis (De Geyter et al. 2012; Patra et al. 2017). Since the signalling deployed by jasmonates and by auxins competes for the protein Auxin Resistant 1 (AXR1), a subunit of the neddylation complex that is needed to activate SCF-type E3 ubiquitin ligases (Schwechheimer et al. 2002), activation of jasmonate signalling will restrain auxin signalling and, thus, cell proliferation. Cell proliferation and alkaloid production have been shown to be negatively correlated in *Datura* cell cultures (Lindsey and Yeoman 1983). Therefore, treatments of cell cultures with JA trigger a two-way impact in favour of alkaloid biosynthesis. Besides its regulation of cascade of genes involved directly in the biosynthesis pathway, it also stimulates the transition to cellular competence enabling alkaloid production and accumulation. In the current study, we saw a stimulating effect of jasmonates, especially in strain C4, where jasmonic acid more than doubled the catharanthine content after 7 days (Fig. 7a). This stimulation declined somewhat during the late stationary phase (day 10), which might be due to toxic side effects of catharanthine, similarly to those that are the base for their therapeutic use in cancer cells (Patra et al. 2017).

We wondered whether penetration into the cell agglomerates might be limiting and therefore tested the volatile derivative methyl jasmonate (MeJA), which in plants is responsible for the systemic spread of stress signalling and can activate the synthesis of vinca alkaloids in plants, cell cultures, rootless shoots, and hairy roots (Aerts et al. 1994; Guo et al. 2013; Vázquez-Flota et al. 2009; Ruiz-May et al. 2009). Again, the effect of MeJA involves upregulation of various genes in their biosynthetic pathway or regulators thereof (van der Fits et al., 2000; Goklany et al. 2009; Simkin et al. 2013). Indeed, we observed that MeJA was more effective than JA, especially in C4 with its more compact morphology. Here, catharanthine was accumulated to amounts that were more than an order of magnitude higher than in the C1 strain (Fig. 7b). Minute amounts of tabersonine were also detected in both the strains. However, neither jasmonic acid nor MeJA was able to induce vindoline pathway. In seedlings of *Catharanthus*, MeJA induces transcripts of *tdc*, *str*, *d4h*, and *dat* (Aerts et al. 1994; Wei 2010), i.e. also the two transcripts *d4h* and *dat* that remain silent in our cell strains (Fig. 5). While we were able to induce *dat* in C4 using MeJA elicitation, *d4h* did still not accumulate (Fig. 7d), which might be the ultimate reason why vindoline cannot be found even in C4 and even after elicitation by MeJA. Instead, elicitation by MeJA strongly induced catharanthine accumulation, which is in good congruence with reports, where UV-B (an inducer of jasmonate synthesis) stimulated catharanthine accumulation in *C. roseus* suspension cells linked with stimulation of tryptophan decarboxylase and strictosidine synthase transcripts (Ramani and Chelliah 2007). These transcripts are well expressed in both of our cell lines (Fig. 5), and therefore are not considered to be the limiting factor. To stimulate them, MeJA will, thus, enhance the formation of catharanthine, but still fail to activate the full potency of the vindoline branch of the pathway (Fig. 1).

Our data, congruent with the literature report, suggests elicitation stimulates the upstream donor pathways but is unable to remove the bottleneck in the vindoline branch. Here, it seems *d4h* remains most reluctant to being persuaded for activation even by MeJA. The failure to partition the donor precursors into the vindoline branch seems to be the reason these cells do not accumulate vinblastine and vincristine.

### Precursor feeding can breach the vindoline bottleneck

As jasmonate elicitation was not able to release the limitation upon the vindoline branch, we attempted precursor feeding. This strategy has been successful to generate pyrroloquinazoline alkaloids in cell cultures of *Adhota vasica* (Sing et al. 2017), or to stimulate the production of artemisinin in *Artemisia annua* suspension cultures (Baldi and Dixit 2008). This strategy has also been applied to cell cultures of *C. roseus* itself: by feeding loganin in combination with MeJA elicitation, it was possible to obtain strictosidine (Fig. 1), an important precursor of catharanthine and vindoline metabolism (El-Sayed and Verpoorte 2002). Likewise, feeding stemmadenine (Fig. 1) yielded tabersonine and catharanthine (El-Sayed et al. 2004). Feeding tabersonine in combination with elicitation by MeJA, we saw a clear stimulation of catharanthine accumulation (Fig. 8b) beyond the level obtained by MeJA elicitation without this precursor (Fig. 7b). Since catharanthine and tabersonine map to different branches of the vinca alkaloid biosynthesis pathway, this result was unexpected, and indicates a feedback activation of tabersonine on the channelling of the common precursor stemmadenine towards the catharanthine branch. This is not the first case where such regulatory feedback loops have been detected. Feeding loganin to a line overexpressing strictosidine synthase resulted in a flux to tryptamine, although this is also located in a different branch of the biosynthetic pathway (Whitmer et al. 2002). In turn, accumulation of tryptamine progressively reduced this flux, indicative of negative feedback by the product. Also, in our experiments, increasing the concentration of tabersonine did not lead to increased amounts of catharanthine (Fig. 8b), indicating product saturation limiting further synthesis of catharanthine.

Since the activity of the vindoline and the catharanthine pathway seems not only to depend on substrate availability but also on regulatory interactions that culminate in suppression of the vindoline branch, we wondered whether feeding of vindoline might deploy subsequent steps of vinca alkaloid synthesis. The fact that the peroxidase gene (*prx1*) required to join the catharanthine and the vindoline moieties was expressed in both strain C1 and C4 (Fig. 5) suggested that feeding vindoline to C4 (which already accumulates decent amounts of catharanthine in response to elicitation) might breach this final hurdle and yield detectable amounts of bisindole alkaloids. In fact, when we combined MeJA elicitation with vindoline feeding in C4 cells, the vincristine pathway was indeed activated and we obtained trace amounts of vincristine (Fig. 8c, d), which is to our knowledge the first time that this has been achieved in cell cultures. However, so far, we were not successful in raising the amount of vincristine by increasing the concentration of the limiting precursor vindoline, although these cells did produce catharanthine and were able to assimilate the externally fed vindoline (Fig. 8b).

### Alkaloid competence correlates with hallmarks of terminal differentiation

The two *Catharanthus* cell strains showed clear differences already on the level of morphology (Fig. 2, Supplementary Fig. S2). These correlated with different growth patterns, whereby fresh weight in C4 increased at twice the velocity than in C1, while the relation was inversed for dry weight (as well as for cell number). Thus, the morphological characteristics clearly indicated that C1 grew by proliferation, while the more pronounced growth of C4 was primarily due to an increase in cell volume. This interpretation was backed up by differences in the appearance of the sub-cellular compartments of these strains. Strain C4 showed a more prominent and larger central vacuole as compared to C1 strain. An expanded vacuole represents a hallmark for the cellular competence to accumulate secondary compounds as exemplarily worked out for phytochrome-triggered anthocyanin accumulation in white mustard (Steinitz et al. 1976; Nick et al. 1993). Sequestration of secondary compounds in the central vacuole also prevents these often toxic compounds from interfering with normal cell metabolism (Wink 1993). For *C. roseus* as well, major alkaloids such as vindoline and catharanthine accumulate in vacuoles as shown by purification and analysis of isolated vacuoles from leaf mesophyll cells (Carqueijeiro et al. 2013).

The importance of the vacuole also implies that cell proliferation and alkaloid production are negatively correlated. In fact, the production of alkaloids is higher during the stationary phase (Roberts 1998). In the current study, we reinforced the transition from proliferation to cell expansion by auxin depletion and were able to promote catharanthine accumulation in strain C4 almost 20-fold. Our findings are in line with other studies, where 2,4-D suppressed the accumulation of TIAs, especially during the growth phase (Arvy et al. 1994), while promoting cell proliferation (Pasquali et al. 1992). Conversely, strain C4 which displayed greater potential to produce alkaloids was also the strain where proliferation activity was lower, while cell expansion was accentuated.

In addition to cell expansion as a general cellular marker for metabolic competence, our data indicate that redox homeostasis plays a crucial role. The cell aggregates of the C4 strain were much larger and compact than those of C1. Oxygen transport models indicated that large aggregates face oxygen depletion at their centre, which was proposed to stimulate secondary metabolic activity as a response to stress (Kolewe et al. 2008). For instance, the production of thiophene increased with increasing aggregate diameter in cell strains from *Tagetes patula* (Hulst et al. 1989). Conversely, in *C. roseus* itself, callus friability correlated with enhanced indole alkaloid production (Zhao et al. 2001). Interestingly, C4 cells host more peroxisomes compared to cells from the C1 strain. Peroxisomes are central to maintain redox balance in plant cells, interacting functionally and physically with mitochondria and with plastids (for review see Pan et al. 2020), for instance through abundant catalases and ascorbate peroxidases (Su et al. 2018). Since catharanthine accumulates in response to exogenous hydrogen peroxide, the higher abundance of peroxisomes in C4 cells along with the higher abundance of catharanthine might report elevated levels of reactive oxygen species in those cells.

This line of thought is also consistent with the different morphology of mitochondria. While, overall, mitochondrial coverage is comparable between both strains and does not change significantly through the culture cycle (Fig. 4c), there are distinct differences in mitochondrial morphology. In C4 cells, vermiform mitochondria prevail (Fig. 4a, b) as they are characteristic for hypoxia (van Gestel and Verbelen 2002). Low partial oxygen pressure disrupts the function of complex III in the mitochondrial electron transport and leads to accumulation of superoxide in the intermembrane space (for review, see Wagner et al. 2018). This deploys retrograde signalling to the nucleus activating stress-related gene expression. The activation of alkaloid accumulation by (hypo)oxidative stress does not mean that this metabolic activity would not require energy. It does—already the sequestration of these alkaloids in the vacuole needs activity of a proton ATPase (Deus-Neumann and Zenk 1984; Carqueijeiro et al. 2013). Such a proton-dependent vacuolar accumulation of alkaloids has also been observed in other plant species (Morita et al. 2009; Otani et al. 2005; Shoji et al. 2009).

A commitment of C4 cells towards terminal differentiation is also indicated by the bundled organisation of actin cables. Actin bundling is an early event in plant programmed cell death (reviewed in Franklin-Tong and Gourlay 2008; Smertenko and Franklin-Tong 2014). This bundling is mechanistically linked with an elevated level of reactive oxygen species (Chang et al. 2015; Eggenberger et al. 2017).

Thus, their larger vacuole, their more expanded appearance, and lower proliferative activity, along with the bundled actin, the vermiform mitochondria, and the more abundant peroxisomes point to a scenario where C4 cells display hallmarks of terminal differentiation that correlate with elevated catharanthine accumulation. To what extent these hallmarks are consequences of hypoxia due to a more compact clustering of the cells remains to be elucidated. The friability of C4 might as well be the consequence of the precocious transition from proliferation to expansion. Overall, these findings about the specific characteristics of these cell strains highlight important cellular markers with high potential to screen population of other *Catharanthus* cell strains with favourable alkaloid production capacity.

### Towards plant cell fermentation of vinca alkaloids

Extensive research has been conducted over the last five decades on the biosynthesis of *C. roseus* TIAs. Tremendous progress in recent years has allowed almost complete profiling of the various steps involved in the vinca alkaloid pathway. Despite these advancements, the current strategies for natural or synthetic production remain unfeasible. Thus, plant cell fermentation represents an encouraging alternative resource for these medicinal compounds. The common view that cells in suspension are just “de-differentiated biomass” is certainly wrong—a closer look usually reveals that they have preserved certain features from their source tissue (Opatrný et al. 2014). In the current work, we have shown that two strains derived from *Catharanthus* seed embryos not only differ in morphology but also with respect to their metabolic competence. This difference consists in a different partitioning of precursors to the catharanthine versus the vindoline branch of TIA biosynthesis. While this finding is encouraging and represents an advance over hairy root or callus cultures that failed to detect vincristine and vinblastine (reviewed in Zhao and Verporte 2007), there is still some way to go. One challenge is, certainly, the upscaling—the successful production of the early precursor ajmalicine from *Catharanthus* suspension cell cultures (Schlatmann et al. 1993) shows that this is feasible, though. The other challenge is the vindoline bottleneck. Specific conditions have to be established to circumvent these metabolic blockades. Quantitative PCRs can be employed to understand the expression of vindoline biosynthetic genes or the transcription regulators of this network. One possibility would be to then overexpress the limiting genes of the vindoline pathway or transcriptional regulators thereof. Although this looks straightforward, the attempts to boost TIA accumulation by overexpression of the jasmonate inducible transcription factor ORCA3 were not successful (Peeble et al. 2009), indicating that the bottleneck might reach beyond the transcriptional level.

In the plant, catharanthine is preferably produced on the leaf surface, while vindoline accumulates in idioblasts in the mesophyll (Almagro et al. 2015). The interaction between different cell types might extend beyond mere passing on of metabolites for downstream processing; there might be regulatory interactions as well that steer the metabolic competence. We have recently addressed this possibility using a modular chip system, where different cell types can interact by a microfluidic flow without the need of physical contact (Finkbeiner et al. 2021). When we placed C1 cells upstream of a chip with C4 cells, we were able to obtain the desired vindoline while simultaneously tabersonine was depleted. Thus, it seems possible to remove the vindoline bottleneck by soluble factors that are generated or released, if the two cell types are allowed to chemically interact. In the future, we will therefore try to replace the vindoline feeding to C4 cells by a combinatorial approach to technically mimic the regulatory interactions of different cell types taking place in a real-world leaf. This shall then help realise the complete potential of these seed embryo–derived *Catharanthus* cell strains.

## Supplementary Information

Below is the link to the electronic supplementary material.

Supplementary file1 (PPTX 196 KB)

Supplementary file2 (PPTX 94 KB)

Supplementary file3 (PPTX 43 KB)

Supplementary file4 (PPTX 96 KB)

Supplementary file5 (PPTX 82 KB)

Supplementary file6 (PPTX 205 KB)

Supplementary file7 (DOCX 20 KB)

## Data Availability

All data used in this research are included in this published article and its supplementary information files.
