# Supplementary figures and images for: Cell type matters: competence for alkaloid metabolism differs in two seed-derived cell strains of Catharanthus roseus
Source: Protoplasma. 2022 Jun 13;260(2):349–69. doi: 10.1007/s00709-022-01781-y (PMC9931846; doi:10.1007/s00709-022-01781-y)

## Slide 1
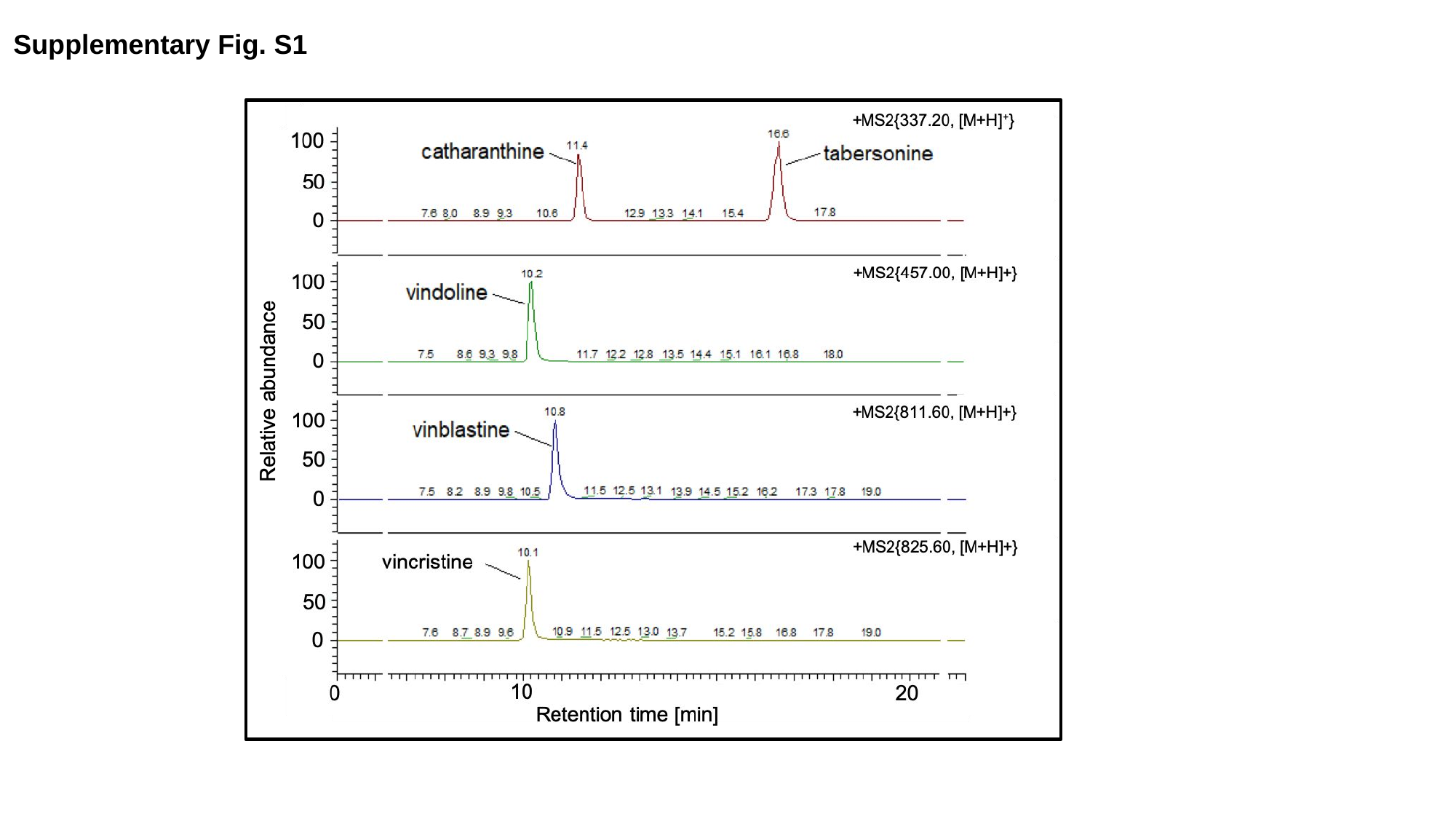

Supplementary Fig. S1

Supplement: Supplementary file 1 — Supplementary file1 (PPTX 196 KB) [file 709_2022_1781_MOESM1_ESM.pptx]

## Slide 1
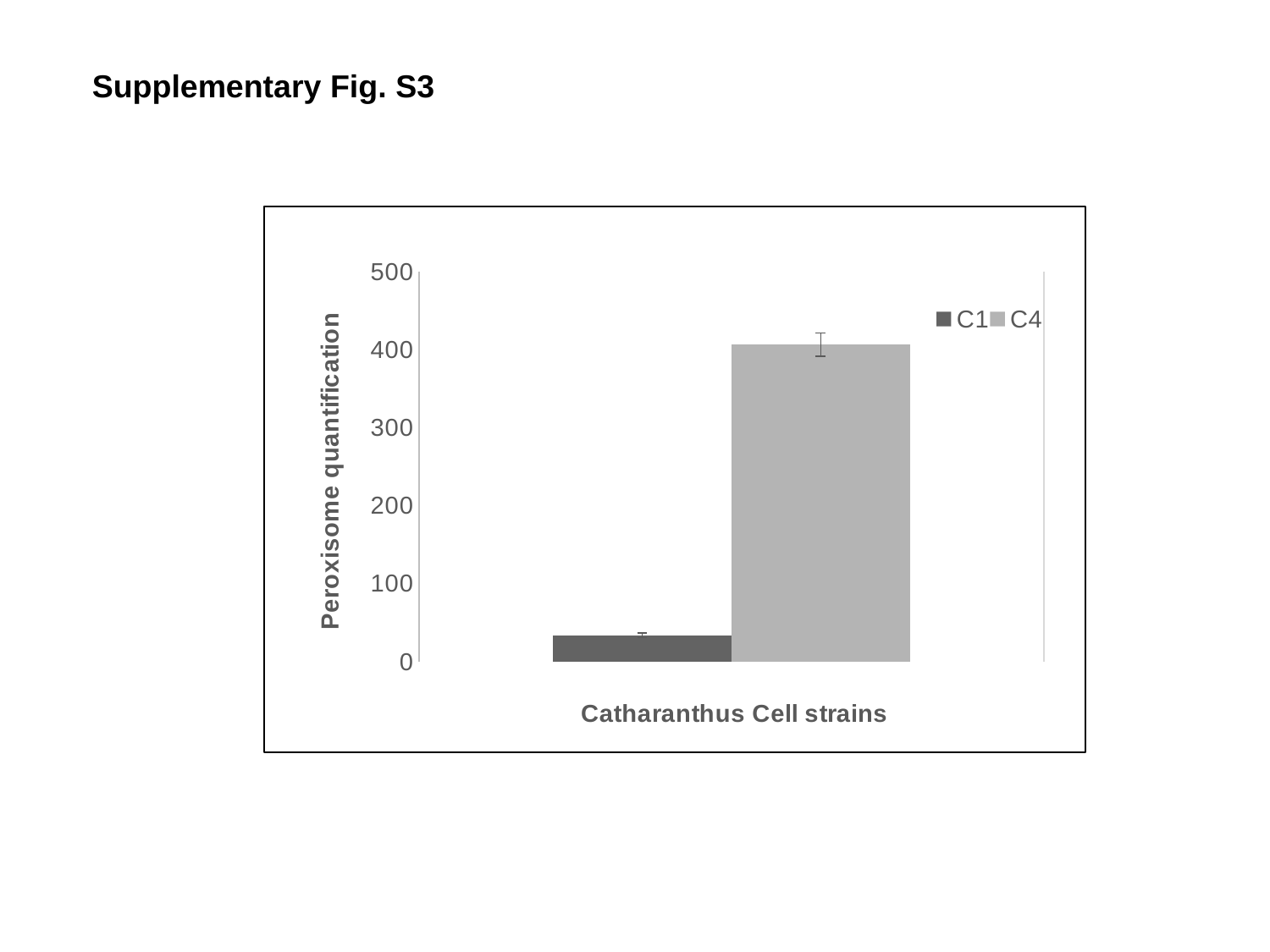

Supplementary Fig. S3
### Chart
| Category | | |
|---|---|---|

Supplement: Supplementary file 3 — Supplementary file3 (PPTX 43 KB) [file 709_2022_1781_MOESM3_ESM.pptx]
