# Supplementary material for: Cell type matters: competence for alkaloid metabolism differs in two seed-derived cell strains of Catharanthus roseus
Source: Protoplasma. 2022 Jun 13;260(2):349–69. doi: 10.1007/s00709-022-01781-y (PMC9931846; doi:10.1007/s00709-022-01781-y)
Supplement: Supplementary file 2 — Supplementary file2 (PPTX 94 KB) [file 709_2022_1781_MOESM2_ESM.pptx]

## Slide 1
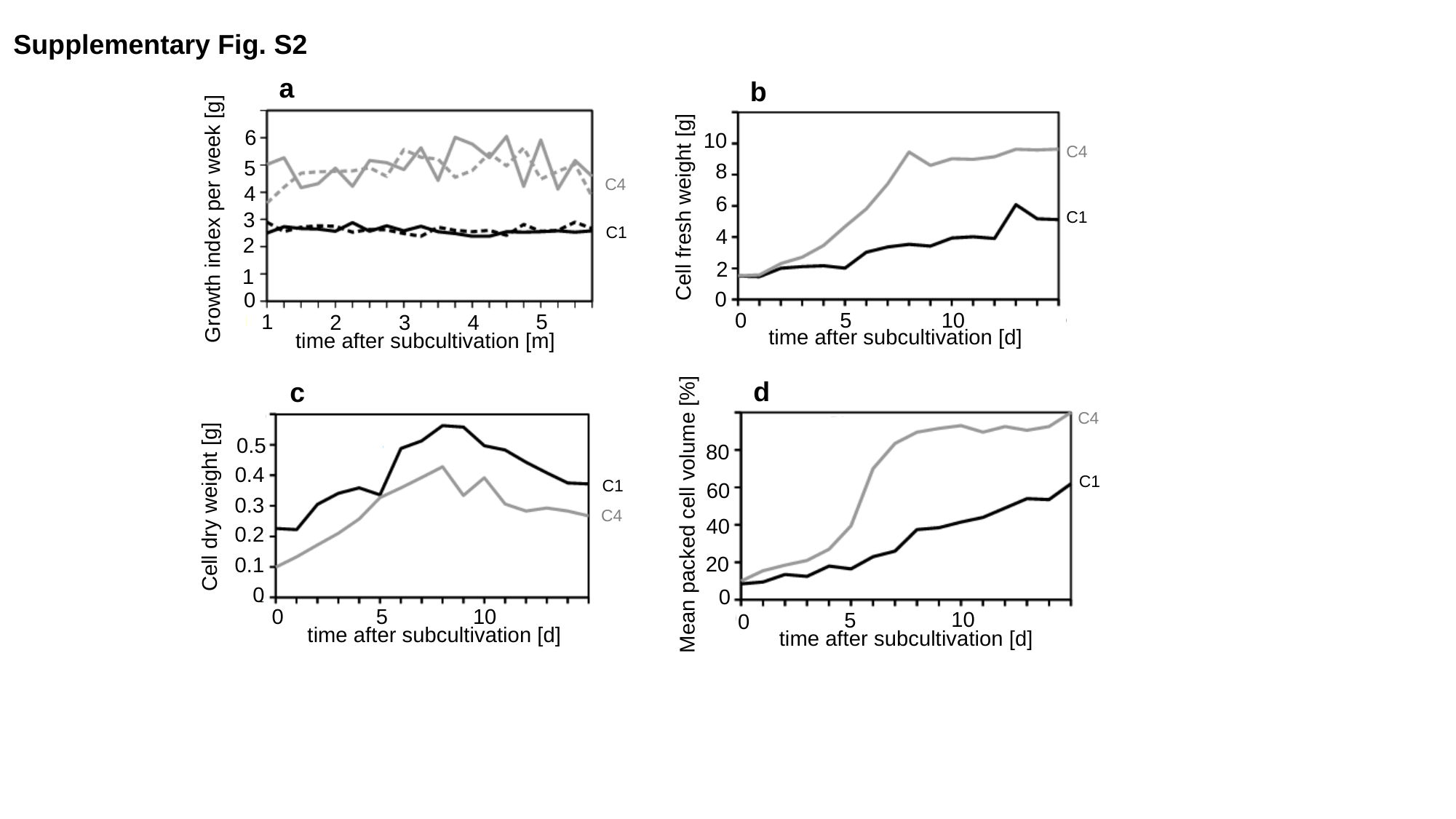

Supplementary Fig. S2
a
b
10
C4
8
6
Cell fresh weight [g]
C1
4
2
0
5
10
0
time after subcultivation [d]
6
5
C4
4
Growth index per week [g]
3
C1
2
1
0
5
1
2
3
4
time after subcultivation [m]
d
C4
C1
10
5
0
time after subcultivation [d]
c
0.5
80
0.4
C1
60
0.3
Cell dry weight [g]
Mean packed cell volume [%]
C4
40
0.2
20
0.1
0
0
10
5
0
time after subcultivation [d]
