# Supplementary material for: Cell type matters: competence for alkaloid metabolism differs in two seed-derived cell strains of Catharanthus roseus
Source: Protoplasma. 2022 Jun 13;260(2):349–69. doi: 10.1007/s00709-022-01781-y (PMC9931846; doi:10.1007/s00709-022-01781-y)
Supplement: Supplementary file 4 — Supplementary file4 (PPTX 96 KB) [file 709_2022_1781_MOESM4_ESM.pptx]

## Slide 1
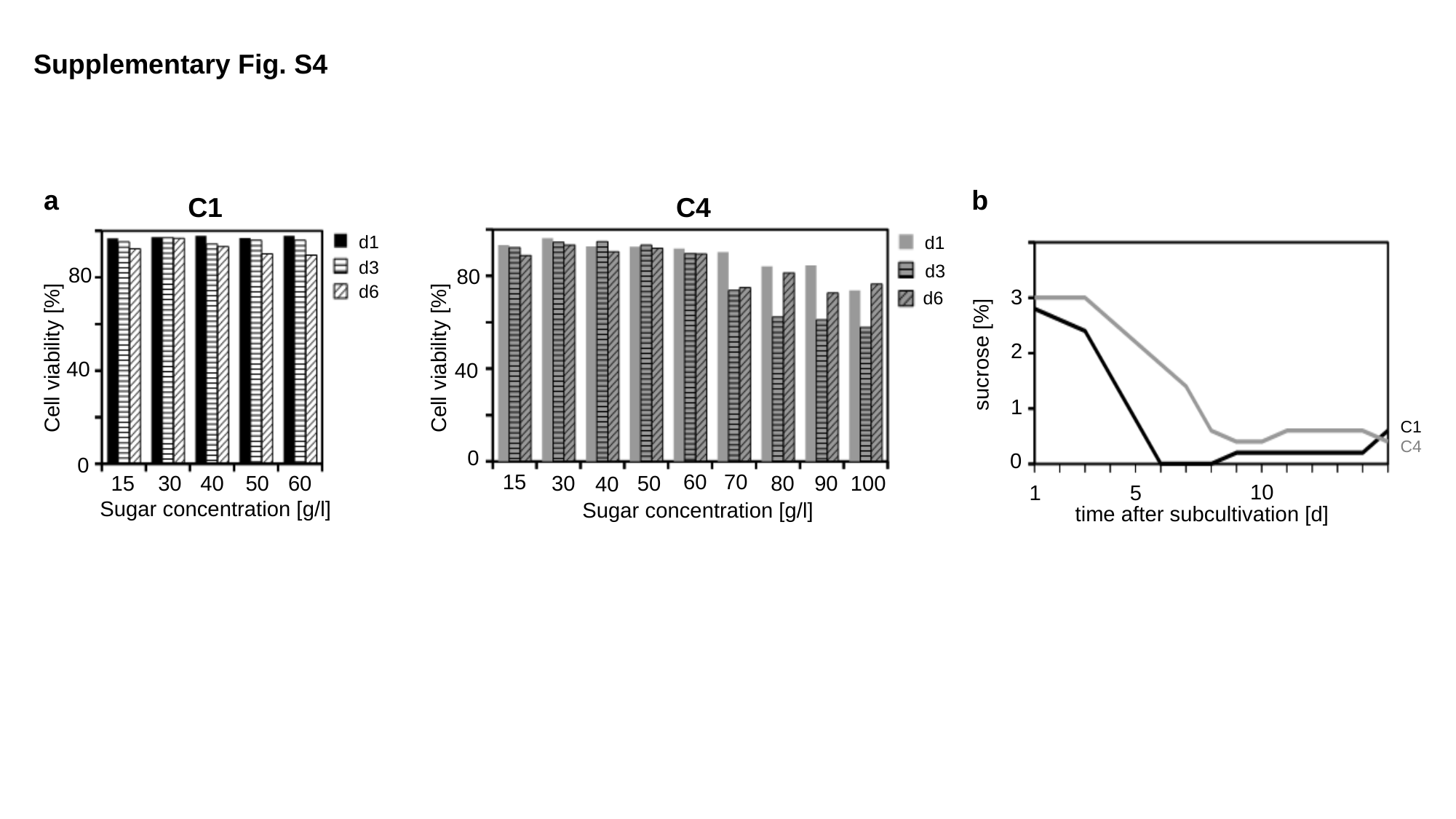

Supplementary Fig. S4
b
a
C1
d1
d3
d6
80
40
0
Cell viability [%]
60
50
30
40
15
Sugar concentration [g/l]
C4
d1
d3
d6
80
40
0
60
15
70
30
80
90
100
50
40
3
2
sucrose [%]
1
C1
C4
0
10
5
1
time after subcultivation [d]
Cell viability [%]
Sugar concentration [g/l]
