# Supplementary material for: Cell type matters: competence for alkaloid metabolism differs in two seed-derived cell strains of Catharanthus roseus
Source: Protoplasma. 2022 Jun 13;260(2):349–69. doi: 10.1007/s00709-022-01781-y (PMC9931846; doi:10.1007/s00709-022-01781-y)
Supplement: Supplementary file 5 — Supplementary file5 (PPTX 82 KB) [file 709_2022_1781_MOESM5_ESM.pptx]

## Slide 1
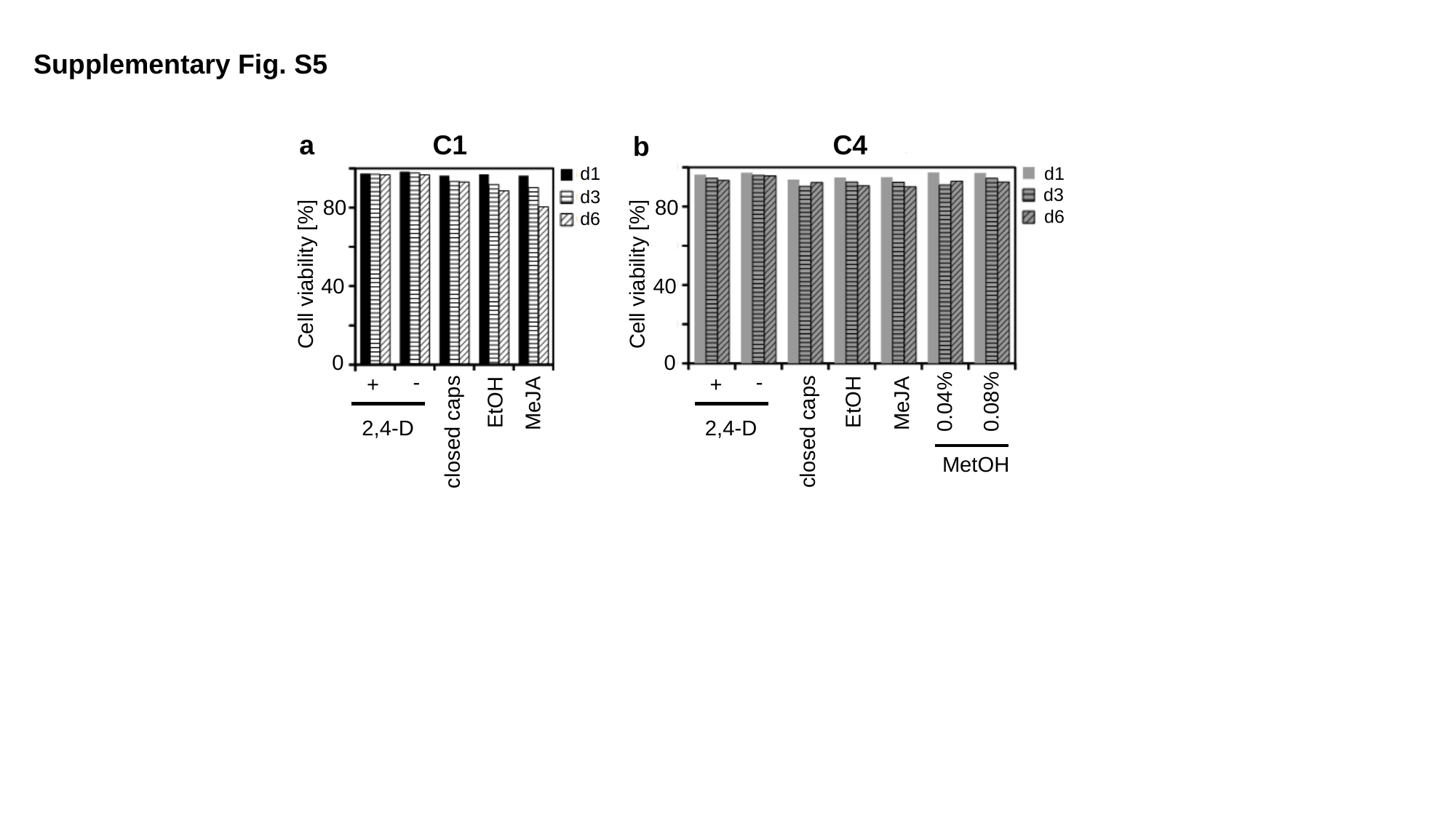

Supplementary Fig. S5
a
C1
C4
b
d1
d3
d6
d1
d3
d6
80
80
Cell viability [%]
Cell viability [%]
40
40
0
0
-
-
+
+
0.08%
0.04%
EtOH
EtOH
MeJA
MeJA
2,4-D
2,4-D
closed caps
closed caps
MetOH
