# Supplementary material for: Cell type matters: competence for alkaloid metabolism differs in two seed-derived cell strains of Catharanthus roseus
Source: Protoplasma. 2022 Jun 13;260(2):349–69. doi: 10.1007/s00709-022-01781-y (PMC9931846; doi:10.1007/s00709-022-01781-y)
Supplement: Supplementary file 6 — Supplementary file6 (PPTX 205 KB) [file 709_2022_1781_MOESM6_ESM.pptx]

## Slide 1
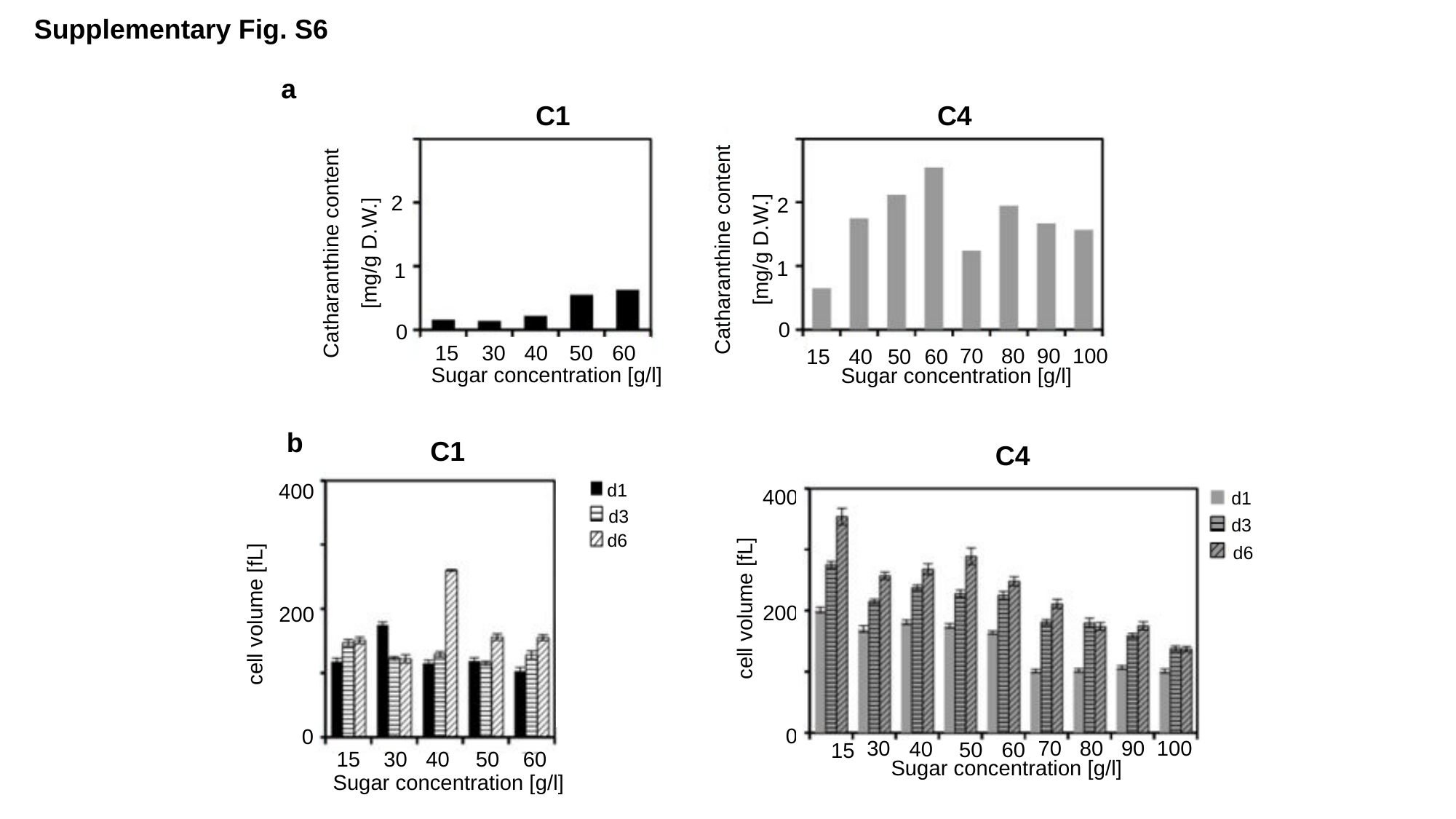

Supplementary Fig. S6
a
C1
C4
2
2
Catharanthine content
[mg/g D.W.]
Catharanthine content
[mg/g D.W.]
1
0
1
0
60
50
30
40
15
70 80 90 100
50
60
40
15
Sugar concentration [g/l]
Sugar concentration [g/l]
b
C1
C4
400
200
0
d1
d3
d6
400
200
0
d1
d3
d6
cell volume [fL]
cell volume [fL]
30
70 80 90 100
 40
50
60
15
60
50
30
40
15
Sugar concentration [g/l]
Sugar concentration [g/l]

## Slide 2
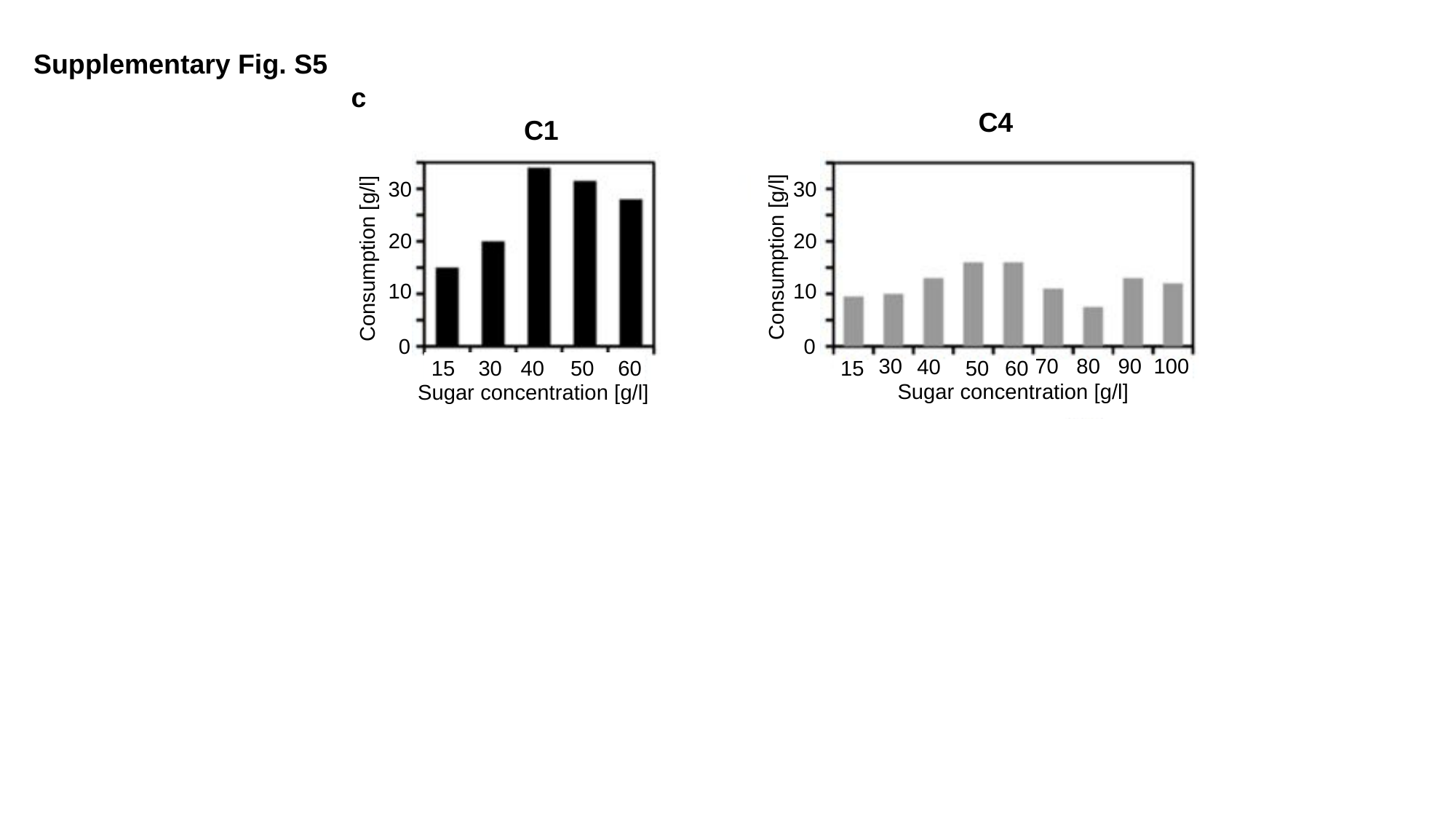

Supplementary Fig. S5
c
C4
C1
30
20
10
0
30
20
10
0
Consumption [g/l]
Consumption [g/l]
30
70 80 90 100
 40
50
60
15
60
50
30
40
15
Sugar concentration [g/l]
Sugar concentration [g/l]
