# Supplementary material for: Cell type matters: competence for alkaloid metabolism differs in two seed-derived cell strains of Catharanthus roseus
Source: Protoplasma. 2022 Jun 13;260(2):349–69. doi: 10.1007/s00709-022-01781-y (PMC9931846; doi:10.1007/s00709-022-01781-y)
Supplement: Supplementary file 7 — Supplementary file7 (DOCX 20 KB) [file 709_2022_1781_MOESM7_ESM.docx]

**Supplementary Material:**

**Title:**

**Cell type matters: competence for alkaloid metabolism differs in two seed-derived cell strains of *Catharanthus roseus*.**

**Authors:**

**Manish L. Raorane^1, 3,*^, Christina Manz^1^, Sarah Hildebrandt^1^, Marion Mielke^1^, Marc Thieme^1^, Judith Keller^2^, Mirko Bunzel^2^, Peter Nick^1^**

**Author information:**

^1^Botanical Institute, Karlsruhe Institute of Technology, Fritz-Haber-Weg 4, D-76131 Karlsruhe, Germany.

^2^Institute of Applied Biosciences, Department of Food Chemistry and Phytochemistry, Karlsruhe Institute of Technology (KIT), 76131 Karlsruhe, Germany

^3^Current address: Institute of Pharmacy, Martin-Luther-University, Halle-Wittenberg, Hoher Weg 8, D-06120 Halle (Saale), Germany.

*Corresponding author: Manish L. Raorane, Institute of Pharmacy, Martin-Luther-University, Halle-Wittenberg, Hoher Weg 8, D-06120 Halle (Saale), Germany, manishraorane@gmail.com

**Supplementary Table S1.** List of chimeric constructs and their targeted subcellular structures

| **Constructs** | **Cellular compartments** | **References** |
| --- | --- | --- |
| FABD2-GFP | actin | Maisch et al. 2009 |
| ST-GFP  Golgi-GFP | Golgi vesicles | Brandizzi et al. 2002 |
| Peroxi-YFP | peroxisomes | Mathur et al. 2002 |
| NtTPC1A-GFP | tonoplast | Qiong L 2014 |

**Supplementary Table S2.** Retention times and MS^2^-fragment ions obtained after fragmentation of pseudo-molecular ions [M+H]^+^ for the vinca alkaloid standard compounds analysed by HPLC-DAD-ESI-MS/MS

| **Standards** | **Retention time (min)** | **[M+H]^+^** | **MS^2^ fragment ions** |
| --- | --- | --- | --- |
| Tabersonine | 16.7 (+/- 0.7) | 337.2 | 305.2, 228.0 |
| Catharanthine | 11.4 (+/- 0.2) | 337.2 | 173.2, 144.1 |
| Vindoline | 10.2 (+/- 0.1) | 457 | 397.5, 188.3 |
| Vinblastine | 10.8 | 811.6 | 751.7, 224.4 |
| Vincristine | 10.1 | 825.6 | 807.5, 765.5 |

**Supplementary Table S3.** Retention times for vinca alkaloid standards analysed with HPLC-DAD

| **Standards** | **Retention time (+/-0.05) [min]** |
| --- | --- |
| Vindoline | 8.81 |
| Vincristine | 10.91 |
| Catharanthine | 12.38 |
| Vinblastine | 16.92 |

**Supplementary Table S4.** List of genes and their primers used in this study

| Genes | Caros accession number | Function | Primer pairs (Sequence 5‘- 3‘) | Product length (bp) | Annealing temperature (° C) |
| --- | --- | --- | --- | --- | --- |
| *exp* | Caros004934.1 | Expressed protein | 5‘ ACAATACCATCGCCATCAC  5‘ AAGAGGACTGCTGGAAGG | 172 | 50 |
| *g10h* | Caros006766.1 | *geraniol 10-hydroxylase* | 5‘ CATTTATTAGGCGACCAACC  5‘GAACTTCTTTCGCCATTGTT | 148 | 50 |
| *as* | Caros016569.1 | *anthranilate synthase* | 5‘ GCGAACATTTGCAGATCCAT  5‘ GGCCGATTTGTTATTGTTCC | 156 | 50 |
| *tdc* | Caros014930.1 | *tryptophan decarboxylase* | 5‘ ATCCGATCAAACCCATACCA  5‘ CGTCATCCTCGACCATTTTT | 143 | 50 |
| *str* | Caros011578.1 | *strictosidine synthase* | 5‘ ACCATTGTGTGGGAGGACAT  5‘ ATTTGAATGGCACTCCTTGC | 152 | 50 |
| *sgd* | Caros009426.1 | *strictosidine β-D-glucosidase* | 5‘ GGAGGCTTCTTGAGTGATCG  5‘ GCAAATTCACCAGTGGCATA | 149 | 50 |
| *t16h1* | Caros001600.1 | *tabersonine 16-hydrolxylase 1* | 5‘ GCCCAAAACAGCCAATATTCAAACC  5‘ ATGTGATGAGTATGGCCACCGC | 154 | 55 |
| *t16h2* | Caros025399.1 | *tabersonine 16-hydrolxylase 2* | 5‘ GATCAACTCACAGTGGCAGTC  5‘ GACTTGAGGACTTGTGATTGGC | 147 | 52 |
| *d4h* | Caros006133.1 | *desacetoxyvindoline 4-hydroxylase* | 5‘ ATAGTTAATCATGGGATTCCACAAGATGTT  5‘ GTTCATGAAACTTACGAACTCCATCTAC | 140 | 52 |
| *dat* | Caros024662.1 | *deacetylvindoline 4-O-acetyltransferase* | 5‘ GGTTTCAATTTATTTCTCACGTAC  5‘ AACTATCAGAAAGGTAAGCATCGA | 150 | 50 |
| *prx1* | Caros007312.1 | *peroxidase 1* | 5‘ AGGGGTGCGATTCATCAGTG  5‘ ATCCTGAAAGCCTGCTGACG | 142 | 54 |
